# Peri-Substituted Acyl Pyrrolyl Naphthalenes: Synthesis, Reactions and Photophysical Properties

**DOI:** 10.3390/molecules30071429

**Published:** 2025-03-24

**Authors:** Junkai Zhao, Robert Pike, Christopher Abelt

**Affiliations:** Department of Chemistry, College of William and Mary, Williamsburg, VA 23185, USA; jzhao12@wm.edu (J.Z.); rdpike@wm.edu (R.P.)

**Keywords:** solvato-chromism, charge transfer, peri, pyrrole, spin–orbit coupling

## Abstract

The preparation of two 1-acyl-8-pyrrolylnaphthalenes (**5** and **6**) and one pyrrolone (**8**) are reported along with the issues complicating the preparations of other compounds. The photophysical behavior of the fused, planar derivative **6** is explored in detail. The fluorescence of **6** shows solvato-chromism due to intramolecular charge transfer in the excited state and enhanced emission in protic solvents. The emission intensity increases very linearly with the H-bond-donating strength of the solvent. Preferential solvation studies, multilinear regression analysis and computational modeling suggest that the fluorescence enhancement results from inhibition of the spin–orbit coupling-promoted intersystem crossing from the π→π* singlet state to an n→π* triplet state. Some of the inhibitions are due to the dielectric stabilization of the excited singlet state. A stronger effect is obtained from H-bonding, which not only further stabilizes the singlet state but also negatively impacts the vibronic coupling between the states.

## 1. Introduction

**Figure 1:**
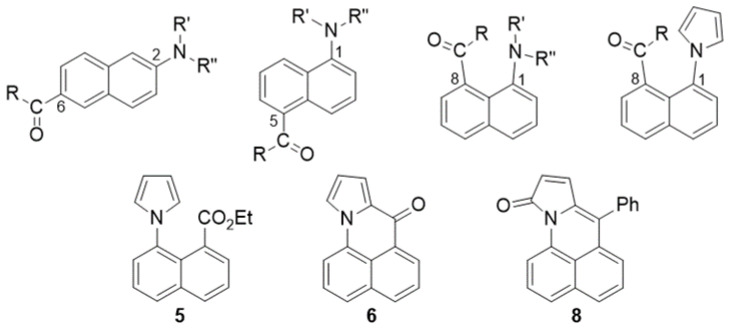
Structures of 2,6-, 1,5- and 1,8-prodan compounds and 1,8-pyrrolyl derivatives (**top** row) and target compounds **5**, **6** and **8** (**bottom**).

Naphthalenes bearing an amine donor and a carbonyl acceptor typically show strong fluorescence due to intramolecular charge transfer (ICT) in the excited state. The most studied member of this group is 6-propionyl-2-(dimethylamino)naphthalene (2,6-prodan) [1]. Other members of this group include the 1,5- and 1,8-regioisomers (Figure 1). Emission likely occurs from an ICT excited state geometry, where the donor and acceptor substituents are co-planar with the naphthalene ring [2,3,4]. Co-planarity is favored even with the 1,8-regioisomers, where the peri-interaction forces the groups to twist in the ground state.

**Scheme 1:**
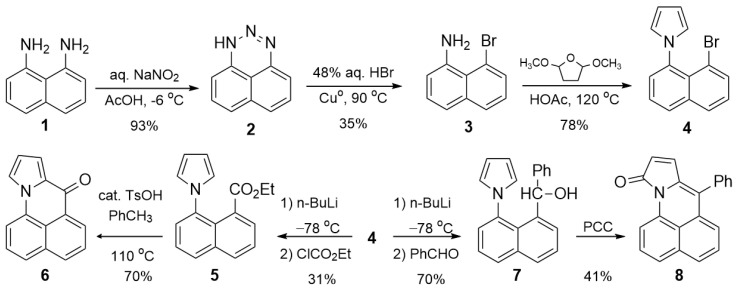
Preparation of **6**–**8** from **1**.

We were curious about the effect of changing the amine group to a pyrrole in the 1,8-regioisomer. The unsaturation in the pyrrole should diminish the peri-interaction strain. The attraction between the nitrogen and the adjacent carbonyl seen with amines [5] should also be diminished. Finally, the aromatic character of the pyrrole should affect the degree of intramolecular charge transfer in the excited state. This paper describes efforts toward preparing 1,8-naphthalenes bearing acyl and pyrrolyl substituents (Scheme 1). The photophysical behavior of compound **6** is explored in detail due to its unusual turn-on fluorescence with protic solvents.

## 2. Results and Discussion

### 2.1. Synthesis and Characterization of Pyrrole Derivatives

#### 2.1.1. Synthesis of Pyrrole Derivatives

Compound **6** is prepared in five steps from 1,8-diaminonaphthalene **1** (Scheme 1). This compound was prepared previously in seven steps from 1-aminonaphthalene as part of a study making analogs of Mitomycin C, an antitumor chemotherapeutic agent [6]. Diazotization of the diamine **1** gives the triazine **2**, which reacts with HBr and activated Cu^o^ to give bromonaphthyl amine **3** [7,8,9]. Variable amounts of naphthyl amine accompany this substitution reaction. This impurity is easily removed by recrystallization from ethanol. The pyrrole was constructed via Clauson–Kaas synthesis, providing the bromopyrrolylnaphthalene **4** as the gateway to all downstream derivatives. In all but one case, further elaboration is via the aryl lithium, resulting from lithium–halogen exchange with n-BuLi. Because the pyrrole group blocks the aryl carbanion, H-atom abstraction is greatly favored over nucleophilic addition. Only electrophiles without acidic hydrogens would avoid H-atom abstraction, and many of these reactants still gave poor yields or mixtures of products.

Ultimately, only ethyl chloroformate and benzaldehyde gave reasonable yields of addition. Other electrophiles that did not afford good results were pivaloyl chloride, ethyl formate, paraformaldehyde and carbonyldipyrrole. Nucleophilic substitution with ethyl chloroformate gives ester **5** in 55% yield after chromatographic purification. This ester proved resistant to further reactions, except for electrophilic aromatic substitution with the pyrrole. The intramolecular cyclization required acid catalysis (TsOH) and gave **6** in 70% yield. Nucleophilic addition reactions of **5** with n-BuLi, DIBAL-H and LiAlH_4_ gave no reaction at low temperatures. The reaction with LiAlH_4_ at high temperatures (60 °C) gave slow conversion to an intractable mixture. In contrast, the nucleophilic addition of the aryl lithium with benzaldehyde gives phenylmethanol **7** in 70% yield. Oxidation of this secondary alcohol gave cyclization with pyrrole moiety, with concomitant oxidation of a pyrrole carbon giving lactam **8**. Different oxidants, including Jones reagent, MnO_2_ and Dess–Martin periodinane, were used to try to limit oxidation to the benzylic carbon. All gave lactam. The yield using pyridinium chlorochromate was 41%. We also tried reacting **7** with protic acids (TsOH, polyphosphoric acid) to see if they would lead to electrophilic substitution as with **5**. These transformations were likewise ineffective. Finally, Rosenmund–von Braun cyanation of **4** gave a mixture containing **6** as the major component.

#### 2.1.2. X-Ray Structures and Select NMR Data

**Figure 2:**
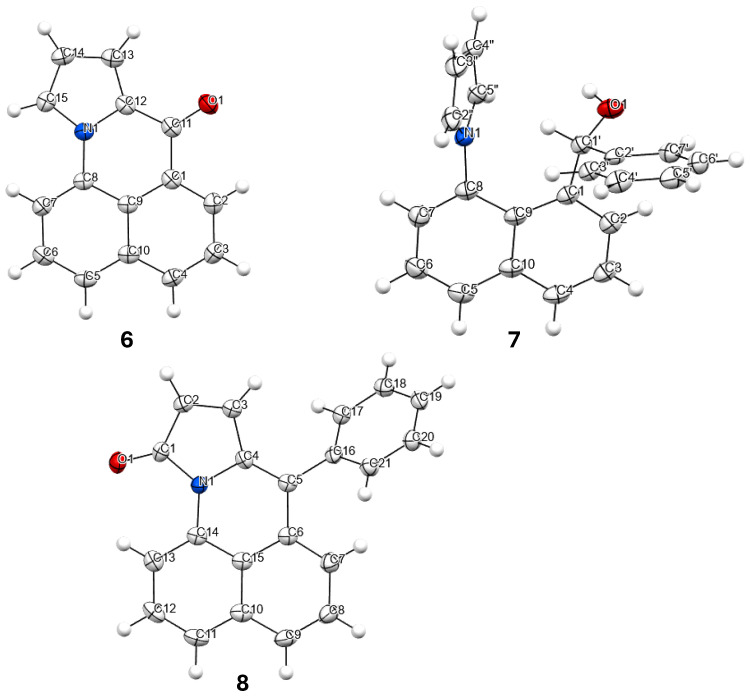
X-ray structures of **6** (**top left**), **7** (**top right**) and **8** (**bottom**). Atom, color: H, white; C, gray; N, blue; O, red. Ellipsoids shown at the 50% level.

**Figure 3:**
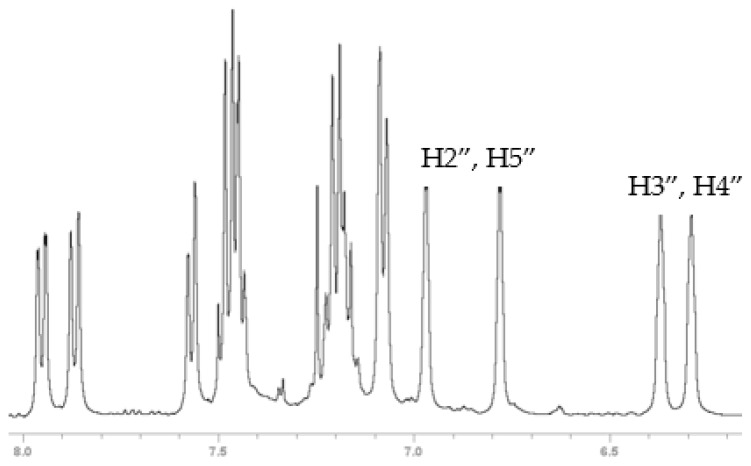
Partial ^1^H NMR spectra of **7** showing the four chemically distinct pyrrole H-atoms.

Compounds **6**, **7** and **8** formed crystals upon slow evaporation of a dichloromethane solution and gave resolved crystal structures (Figure 2). As would be expected, ketone **6** is planar. The pyrrole ring is slightly canted toward the carbonyl (C15-N-C8 = 128°, C12-N-C8 = 124°). Lactam **8** is also mostly planar. The phenyl ring is twisted out of the molecular plane by 51°. While **7** is only a synthetic intermediate, it has the most interesting crystal structure. The free pyrrole ring is twisted ¾ of the way to being perpendicular (67°) to the naphthalene. The pyrrole is canted away slightly from the phenylmethanol group (C9-C8-N = 122°), but the phenylmethanol group is canted significantly from the pyrrole (C9-C1-C1′ = 126°). The C1′ methine H is pointed towards the center of the pyrrole ring, and it lies in the naphthalene plane. The C′ methine is a chiral center. In Figure 2, it is in the *R*-configuration. One interesting consequence of this chirality is that the otherwise equivalent H-atoms on the pyrrole (H2″, H5″ and H3″, H4″) become diastereotopic due to the axial chirality induced by the hindered rotation about the C8-N bond (Figure 3). Likewise, the four pyrrole carbon atoms become distinct so that the ^13^C spectrum shows 19 carbon atoms instead of 17 (Figure S4).

### 2.2. Photophysical Studies

Of the three chromophores, ketone **6** was characterized in detail while the properties of ester **5** and lactam **8** were examined briefly because neither was very solvato-chromic.

#### 2.2.1. Absorption

**Figure 4:**
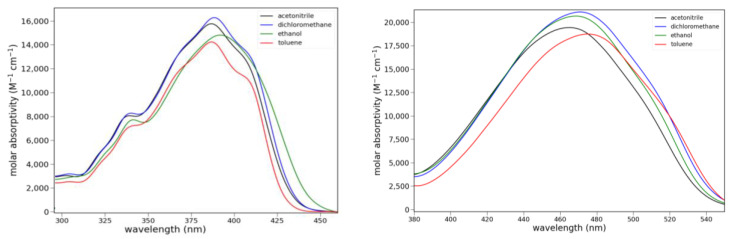
Absorption spectra of **6** (**left**) and **8** (**right**) in various solvents.

The long-wavelength absorption maxima for **6** are at 389 ± 3 nm over a series of solvents ranging from apolar, aprotic to polar, protic (toluene, dichloromethane, acetone and ethanol). Lactam **8** shows much longer wavelength absorptions at 470 ± 5 nm over the same series of solvents. Ketone **6** absorbs slightly less strongly than **8**, and it shows vibronic bands. Absorption spectra for **6** and **8** are shown in Figure 4, and the absorption data are compiled in Table S1.

#### 2.2.2. Fluorescence

**Figure 5:**
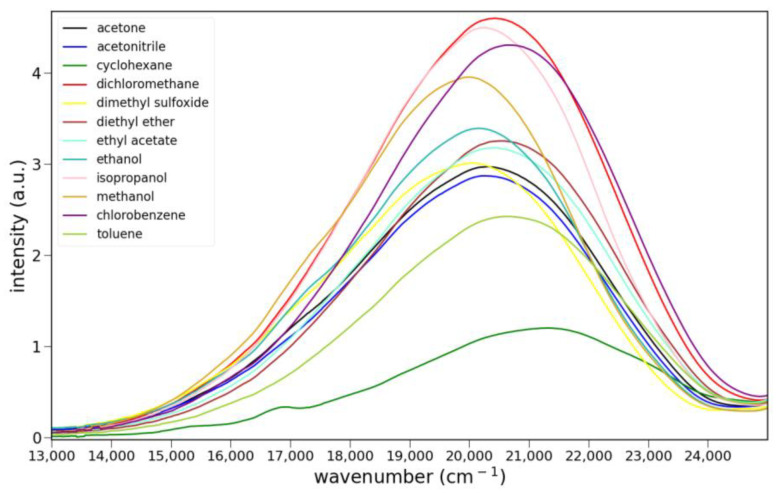

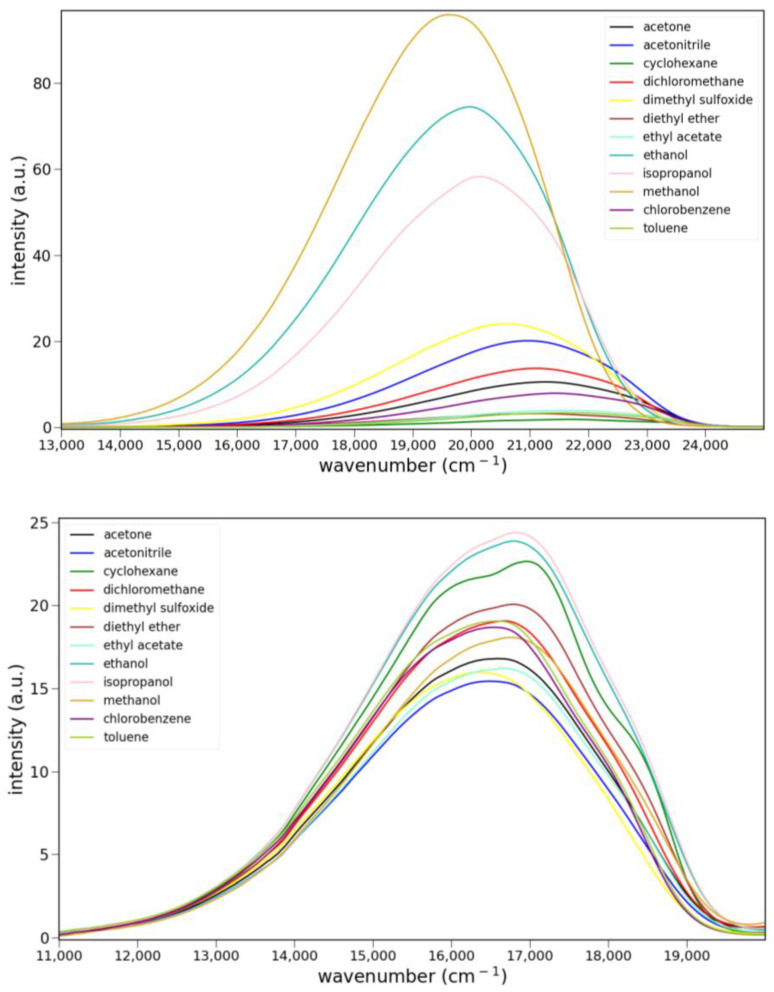
Fluorescence spectra of 7.9 × 10^−5^ M **5** (**top**), 9.6 × 10^−6^ M **6** (**middle**) and 1.0 × 10^−5^ M **8** (**bottom**) in various solvents: cyclohexane, toluene, chlorobenzene, methylene chloride, ethyl acetate, diethyl ether, acetone, acetonitrile, dimethyl sulfoxide, isopropanol, ethanol and methanol. Excitation was at 365 nm for **5** and **6** and 455 nm for **8**.

The fluorescence spectra of **5**, **6** and **8** in various solvents are shown in Figure 5. Only ketone **6** shows clear evidence of charge transfer emission. Ester **5** shows some solvato-chromism, while lactam **8** shows almost none. Protic solvents have little effect on the fluorescence of **5** or **8**. However, the emission of **6** is noticeably stronger in isopropanol, ethanol and methanol than all other solvents. The quantum yield for **6** in ethanol was determined to be 0.21 ± 2 using anthracene as a reference (*Φ* = 0.30). The emission maxima and relative integrated intensities are collected in Table S2.

#### 2.2.3. Solvato-Fluorochromism

**Figure 6:**
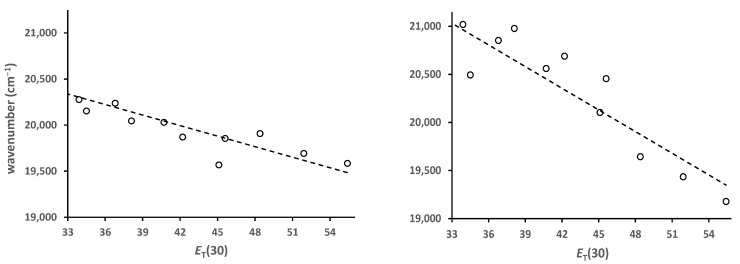
Plots of the emission maxima (cm^−1^) vs. E_T_(30) for **5** (**left**) and **6** (**right**) and lines of best fit, slopes, *R*^2^: −38, 0.77; −75, 0.82. Solvents are toluene, chlorobenzene, dichloromethane, ethyl acetate, diethyl ether, acetone, acetonitrile, dimethyl sulfoxide, isopropanol, ethanol and methanol.

Solvato-fluorochromism results when the excited state has a different dipole moment than the ground state. Solvent molecules will reorient around the excited state in response to the change in charge distribution. If the excited state dipole moment is greater than the ground state, then more polar solvents will better stabilize the excited state, resulting in positive solvato-fluorochromism. As a result, the emission will shift to lower energy. The magnitude of the solvato-chromism can be determined through plots of the fluorescence maxima (in cm^−1^) vs. some solvent polarity parameter. One of the more generally used of these is the E_T_(30) parameter [10]. Plots of emission maxima vs. E_T_(30) are shown in Figure 6 for **5** and **6**. The wavenumber axis is the same for both. A comparison of the two graphs shows that **5** is less solvato-chromic than **6.** The slope of the best-fit line is almost half for **5** (−38 vs. −75). relative to **6.** By comparison, prodan derivatives are much more solvato-chromic and show slopes around −200 [3,4].

#### 2.2.4. H-Bonding Turn-On Fluorescence

**Figure 7:**
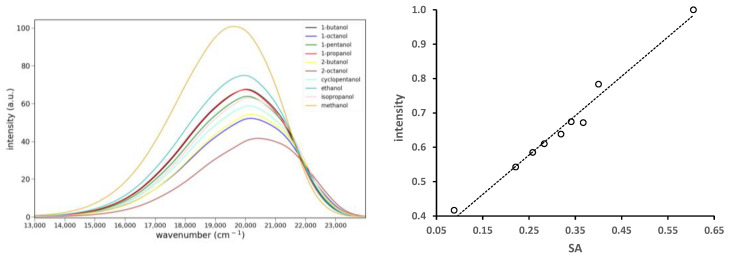
Fluorescence spectra of 1.9 × 10^−5^ M **6** in various protic solvents (**left**). Solvents are 1-butanol, 1-octanol, 1-pentanol, 1-propanol, 2-butanol, 2-octanol, cyclopentanol, ethanol, isopropanol and methanol. Plot of relative integrated fluorescence intensity vs. the SA solvent parameter and the line of best fit (**right**). Slope, *R*^2^: 1.15, 0.98.

The solvato-chromism studies (Figure 5) showed that **6** fluoresces much more strongly in protic solvents. The emission of **6** in other protic solvents is shown in Figure 7 (left). These spectra reveal a regular progression of increasing fluorescence intensity coupled with shifting to lower energy. While the alcohol solvents do not vary greatly in their polarity, they differ in their H-bond donating ability. This solvent property has been quantified by several empirical scales [11]. This paper uses the solvent acidity scale developed by Catalán [12]. A plot of the relative emission intensity vs. Catalán’s SA parameter is shown in Figure 7 (right). The increase in intensity is remarkably linear with respect to this parameter. We have previously reported a linear response with the SA parameter with several prodan derivatives where the carbonyl group is twisted out of plane [13]. In these cases, H-bonding led to fluorescence quenching. Linearity was achieved by correlating the magnitude of the quenching (log_10_(I_o_/I) where I_o_ is the emission intensity in toluene) with the SA parameter. We recently reported one case where protic solvents gave rise to a fluorescence increase [14]. However, the increase was correlated with the presence of a protic solvent, not its H-bond donating ability. Multilinear regression was employed to explore the dependence of the emission intensity on various solvent properties. A delta function (values of 1 or 0) was used to model protic vs. aprotic solvents. The multilinear regression showed that the emission intensity correlated well with the delta function and the solvent dipolarity (SdP), but not the SA parameter.

**Figure 8:**
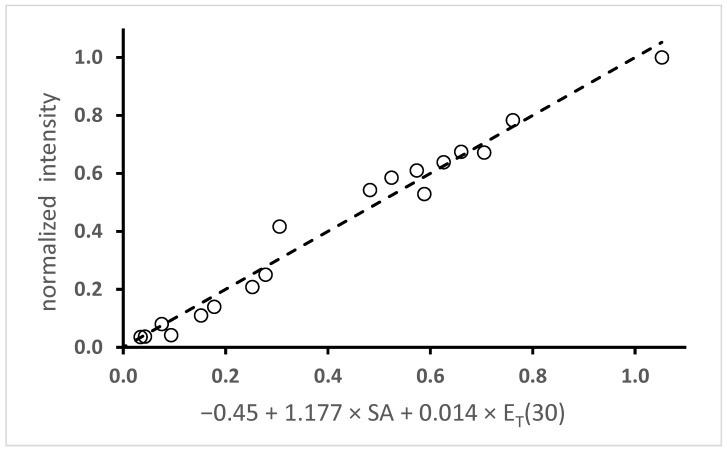
Plot of the normalized fluorescence intensity vs. the value of −0.45 + 1.177 × SA + 0.014 × E_T_(30) and the line of best fit using coefficients from Table 1. The *R^2^* value is 0.98.

The fluorescence intensity of **6** was examined through multilinear regression (Equation (1)). The data from Figure 5 and Figure 6 were combined so that both protic and aprotic solvents were included. The integrated intensities were normalized by dividing by the intensity in methanol. Table 1 shows the results for three correlations with the following combinations of parameters: SA and E_T_(30); SA and SdP; and SA, E_T_(30) and SdP. The *p*-values are shown as log_10_*p*. The correlation with the given variable is considered statistically relevant (i.e., the null hypothesis can be rejected) when the log_10_*p* values are −1.3 or less (*p* < 0.05). The first entry is the most reasonable. Not only is the *F*-value the greatest, but also both variables pass the *p*-test. The second entry fails because the SdP parameter does not pass the *p*-test. While the third entry passes the *p*-test for all three variables, the *F*-value is smaller than the first entry. More importantly, the coefficient for the SdP parameter is negative, which is nonsensical because this would indicate that the fluorescence intensity decreases with increasing polarity, but the opposite is observed. Both the regression coefficient and the parameter range determine which of the parameters is the most important in the correlation. The SA parameter varies from 0 to 0.605 while the E_T_(30) varies from 30.9 to 55.4. Multiplying the range for these parameters and the regression coefficient gives values of 0.71 for the SA parameter and 0.34 for the E_T_(30) parameter. This result indicates that the SA parameter is about twice as important as the E_T_(30) parameter in the correlation. Finally, the results of the best-fit linear regression are shown in Figure 8.

*I/I*_MeOH_ = *c*_0_ + *c*_1_ × SA + *c*_2_ × E_T_(30) + *c*_3_ × SdP (1)

#### 2.2.5. Preferential Solvation

Preferential solvation studies have offered insights into the mechanism of H-bonding interactions with excited states that involve carbonyl groups. In binary solvent mixtures containing a protic solvent and an aprotic solvent, the excited state might be solvated preferentially by the protic component if the carbonyl oxygen becomes more negatively charged. The one- and two-step solvent exchange processes developed by Rosés and Bosch are shown in Equations (2) and (3) [15,16,17,18,19,20,21,22]. In these expressions, F* is the first singlet excited state, RH is the aprotic component, ROH is the protic component and the solvent in parentheses is in the cybotactic region surrounding the solute. The constants *f*_1/0_ and *f*_2/0_ quantify the single- and double-exchange processes, respectively. Both the quantum yield (*Φ*) and the product of the quantum yield and fluorescence center of gravity (*Φ* × ῦ_cog_) are used to characterize preferential solvation. The fractional change in these quantities, *Γ*_1_ and *Γ*_2_, is calculated as shown in Equations (4) and (5), where the subscripts 0 and 2 refer to the values in pure aprotic and protic solvent, respectively. The wavenumber center-of-gravity (ῦ_cog_) is determined through Equation (6), where *I* is the fluorescence intensity.

(2) $$F^{*}\left(RH\right)+ROH⇄F^{*}\left(ROH\right)+RH\;single\;exchange:f_{1/0}$$

(3) $$F^{*}\left(RH,RH\right)+2\;ROH⇄F^{*}\left(ROH,ROH\right)+2\;RH\;double\;exchange:f_{2/0}$$

(4) $$\Gamma_{1}=\left(\Phi_{0}-\Phi \right)/\left(\Phi_{0}-\Phi_{2}\right)$$

(5) $$\Gamma_{2}=\left(\Phi_{0}\times {\tilde{\upsilon }}_{0_{cog}}-\Phi \times {\tilde{\upsilon }}_{cog}\right)/\left(\Phi_{0}\times {\tilde{\upsilon }}_{0_{cog}}-\Phi_{2}\times {\tilde{\upsilon }}_{2_{cog}}\right)$$

(6) $${\tilde{\upsilon }}_{cog}=\left(\Sigma I\times \tilde{\upsilon }\times \Delta \tilde{\upsilon }\right)/\left(\Sigma I\times \Delta \tilde{\upsilon }\right)$$

**Figure 9:**
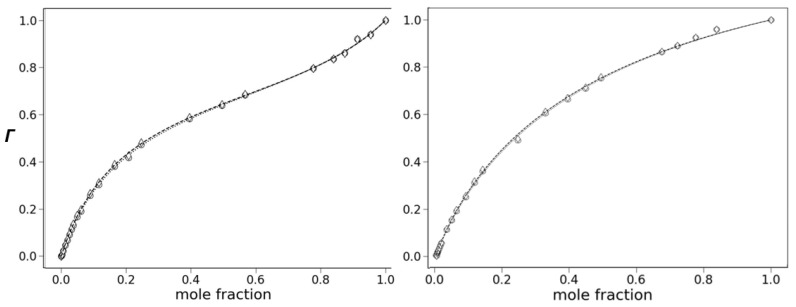
Plots of *Γ*_1_ (o, ^….^) and *Γ*_2_ (◊, - - -) vs. methanol mole fraction. Left: toluene, right: acetonitrile. The lines are the best fit for Equation (7).

Preferential solvation is revealed through plots *Γ*_1_ and *Γ*_2_ vs. protic solvent mole fraction (χ_i_). A linear response (*Γ*_1_ = χ_i_ and *Γ*_2_ = χ_i_) indicates the absence of preferential solvation, while deviation from this line indicates that it is occurring. Plots of *Γ*_1_ and *Γ*_2_ vs_._ mole fraction of methanol for the fluorescence of **6** are shown in Figure 9. The aprotic component is toluene for the left plot and acetonitrile for the right. Both plots show an upward deviation from linearity as the protic component increases, indicative of preferential solvation. The deviation is caused by the increase in quantum yield due to the overabundance of methanol in the cybotactic region compared to the bulk composition. Both *Γ*_1_ and *Γ*_2_ give the same response, and these values are almost identical. This result indicates that changes in quantum yield and emission center-of-gravity have the same mechanistic origin. We have reported cases where there is a divergence in *Γ*_1_ and *Γ*_2_ plots due to strong H-bond quenching that is mechanistically distinct from energy-gap quenching [23].

The preferential solvation can be analyzed through Equation (7) using the constants in Equations (2) and (3). The constant *r* is defined as (*Φ*_0_ − *Φ*_1_)/(*Φ*_0_ − *Φ*_2_) for *Γ*_1_ and (*Φ*_0_ × ῦ_cog0_ − *Φ*_1_ × ῦ_cog1_)/(*Φ*_0_ × ῦ_cog0_ − *Φ*_2_ × ῦ_cog2_) for *Γ*_2_. This constant represents the fractional change for singly H-bonded species. The values for *f*_1/0_, *f*_2/0_ and *r* are established through non-linear least-squares fitting of the plot of *Γ* vs. mole fraction and are shown in Table 2. The dashed lines in Figure 8 are the best-fit curves using the determined values.

*Γ* = (*f*_2/0_ × χ_i_^2^ + *f*_1/0_ × *r* × (χ_i_ − χ_i_^2^))/((1 − χ_i_)^2^ + *f*_2/0_ × χ_i_^2^ + *f*_1/0_ × (χ_i_ − χ_i_^2^)) (7)

The results in Table 2 reveal several interesting features. Firstly, the similarities in the values for *Γ*_1_ and *Γ*_2_ corroborate the conclusion that H-bonding and solvent polarity have the same mechanistic effect on fluorescence. Secondly, the results with acetonitrile indicate that H-bonding, apart from solvent polarity, is largely responsible for the effect on the fluorescence intensity. Acetonitrile and methanol have similar SdP values: 0.974 and 0.904, respectively. In fact, increasing the methanol fraction should slightly decrease the bulk polarity. As a result, the observed increase in the quantum due to preferential solvation must be due to H-bonding.

### 2.3. Computational Studies

**Figure 10:**
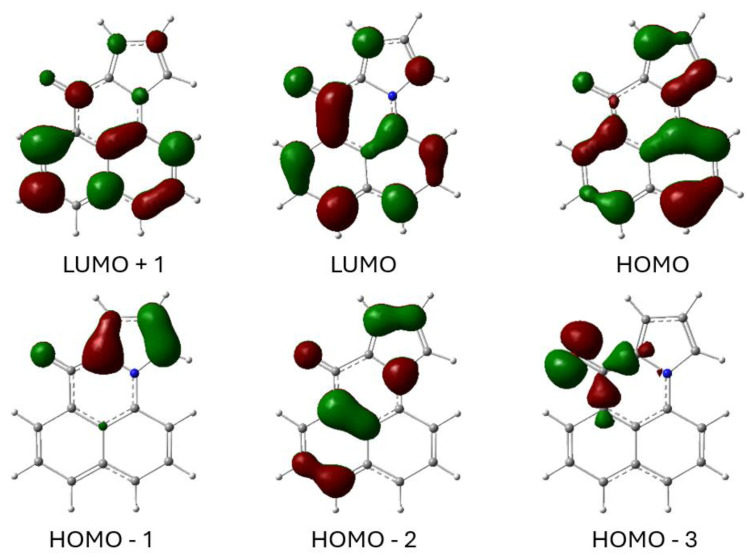
Calculated frontier molecular orbitals for the first singlet excited state of **6** using the DFT CAM-B3YLP method with the 6-311G+(2d,p) basis set and the IEFPCM solvent model for acetonitrile. Atom, color: H, white; C, gray; N, blue; O, red. Molecular orbital phases are dark red and green.

The optimized electronic structures of **6** in the ground and excited state were calculated using Gaussian 16 [24]. The frontier molecular orbitals of **6** for the acetonitrile-solvated first singlet excited state (S_1_) are shown in Figure 10. The LUMO is largely the in-phase combination of the naphthalene B_2g_ LUMO and the carbonyl π* orbital. The HOMO is the out-of-phase combination of the naphthalene A_u_ HOMO and a pyrrole HOMO. The HOMO-1 is mostly the other pyrrole HOMO orbital. The lowest energy singlet excited state results from a simple HOMO→LUMO transition. Because the HOMO involves the pyrrole N electrons and the LUMO involves the carbonyl π* orbital, there is some intramolecular charge transfer from the N to the carbonyl O. The HOMO-3 orbital is included in the figure because it is the in-plane, non-bonding orbital characteristic of the carbonyl group.

The calculations offer an explanation for the origin of the turn-on fluorescence behavior of **6** seen with protic solvents. Excited carbonyl compounds often give little to no fluorescence due to rapid intersystem crossing to the triplet state facilitated by spin–orbit coupling. In the current case, the excitation is π→π*. Intersystem crossing would require a n→π* triplet state of similar energy according to the El-Sayed rules. The second to last row of Table 3 shows that the initially formed excited singlet state is calculated to be just higher energy than a n→π* (HO-3→LU) triplet state. Relaxation of the excited state shifts the singlet state to lower energy (last row). The stabilization of the singlet is dependent on the solvent. Acetonitrile lowers the energy to a greater extent than toluene. The solvato-chromic behavior in Figure 5 shows that the fluorescence of **6** is much stronger in acetonitrile than in toluene. Thus, an explanation for the increased quantum yield is that solvent can modulate the energies of the π→π* singlet state and the n→π* triplet state and affect the rates of intersystem crossing that lead to deactivation. While polarity clearly has an impact on the fluorescence intensity, the stronger effect is due to H-bonding.

Calculations on a H-bonded model were carried out to see what effect H-bonding has on the system. Methanol was chosen as the H-bond donor, and a toluene dielectric continuum model was used to approximate the preferential solvation results that forecast the emission energy of a singly H-bonded excited state in a solvent shell of toluene. The lowest energy H-bonded excited state was determined by allowing optimization of various OH - - - O = C distances at a lower level of theory (6-31G+). This procedure gave a distance of 1.72 Å, and the system was optimized at the higher level (6-311G+(2d,p)) at this fixed distance. Table 3 shows that the H-bonded complex has a lower relaxed singlet excited state energy than with toluene alone by 1.5 kcal/mol, but not as low as with acetonitrile alone (Δ = 2.7 kcal/mol). However, the energy difference between the relaxed singlet and the n→π* triplet state is smaller (10.5 vs. 14.3 kcal/mol).

The predicted absorption and emission maxima are shown in Table 4 along with the experimental values. While the calculations reproduce the solvent trends, they overestimate the absorption energy and underestimate the emission energy. As a result, the predicted Stokes shifts are double the observed values. The change in the partial charges on the O and N atoms (Table 4) going from the ground state to the excited state confirms the intramolecular charge transfer character of the excited state. Interestingly, one effect of H-bonding is to generate greater charge transfer between these atoms than with the acetonitrile model.

The calculations suggest that the solvent stabilization of the excited singlet state contributes to the increase in the fluorescence quantum yield. However, it cannot be the only factor. If it were, then the increase in quantum yield should correlate more strongly with a polarity parameter and not a H-bond donating ability parameter (SA). Returning to Table 1, the best correlation for the fluorescence intensity is achieved with a combination of SA with E_T_(30). In fact, the latter parameter is not a purely polarity scale. It has a H-bonding component, as shown by the differing values for acetonitrile (45.6) vs. methanol (55.4). Of the two parameters, the correlation is stronger with the SA parameter, given the better *p*-value and greater contribution to the fit (vide supra). A multilinear regression analysis of the ῦ_cog_ values using Equation (8) is shown in Table 5. In contrast to the previous regression analysis, the correlations are not as strong. The correlation with E_T_(30) or SA gives the best *F*-values. Surprisingly, combining parameters gives worse *F*-values. However, combining two polarity parameters, SdP and E_T_(30), is slightly better than combining E_T_(30) with SA.

ῦ_cog_ = *c*_0_ + *c*_1_ × SA + *c*_2_ × E_T_(30) + *c*_3_ × SdP (8)

## 3. Materials and Methods

The reagents were obtained from Acros Organics or Sigma-Aldrich (Saint Louis, MO, USA) unless otherwise indicated. Proton and carbon nuclear magnetic resonance spectra were obtained with an Agilent DD2-400 spectrometer (Santa Clara, CA, USA). X-ray measurements were made using a Bruker-AXS D8 Venture four-circle diffractometer (Billerica, MA, USA), equipped with a microfocus Mo tube and a Photon 3 CPAD detector. All solvents used for absorption and fluorescence were spectrophotometric grade. Absorption and fluorescence data were collected using a fiber optic system with an Ocean Optics Maya CCD detector using a miniature deuterium/tungsten lamp and a 365 or 455 nm LED light source, respectively. Cuvettes were thermostated at 23 °C for fluorescence studies. Emission intensities were processed by subtracting the electronic noise, converting wavelengths to wavenumbers, multiplying by λ^2^/λ_max_^2^ to account for the effect of the abscissa-scale transformation [25] and dividing by the spectral response of the Hamamatsu S10420 CCD (Saitama, Japan). Relative quantum yields were determined using anthracene as the reference (*Φ* = 0.30) [25], using the method of standard additions.

Electronic structure calculations were carried out using Gaussian 16 [24]. Ground-state geometries were optimized using the DFT CAM-B3YLP method with the 6-311G+(2d,p) basis set and the IEFPCM solvent model for toluene or acetonitrile. Excited states were optimized using the TD-SCF DFT CAM-B3LYP method with the 6-311G+(2d,p) basis set and the IEFPCM solvent model for toluene or acetonitrile.

Crystals for X-ray diffraction were grown by dissolving the compounds in a minimum amount of dichloromethane and allowing the solvent to evaporate slowly.

### 3.1. 1H-Naphtho [1,8-de][1,2,3]triazine *(**2**)*

Naphthalene-1,8-diamine, **1** (4.16 g, 26.3 mmol) was slurried in acetic acid (46 mL) and water (11 mL). The mixture was cooled to −6 °C with an ice–acetone bath. A solution of NaNO_2_ (1.82 g, 26.4 mmol) in water (11 mL) was cooled in ice water. The sodium nitrite solution was added dropwise to the diaminonaphthalene slurry, and the reaction was stirred for 1 h at −6 °C. The resulting solid was collected with suction and washed with ice-cold water until the filtrate was clear. The solid was left to dry uncovered in a fume hood. This reaction was repeated twice more on the same scale, giving crude **2** (12.44 g, 73.5 mmol, 93%). It was used directly in the next step.

### 3.2. 8-Bromonaphthalen-1-amine *(**3**)*

Copper powder (3.8 g) was activated by stirring with two 50 mL portions of I_2_ (2 g) in acetone (100 mL), decanting the solution carefully after each wash. The residue was further activated by stirring with three 33 mL portions of acetone-conc. aq. HCl (50 mL each), decanting the solution carefully after each wash. Finally, the copper was dried under a stream of Ar. This procedure gave 3.2 g of bright red copper powder. Triazine **2** (5.6 g, 33.1 mmol) was covered with 48% aq. HBr and immersed into an oil bath preheated to 90 °C. Stirring was commenced just before the copper powder (1.6 g, 25.2 mmol) was added in portions. After an induction period of ~one minute, the reaction began to give off bubbles. The reaction was stirred for 5 min at 85 °C after the copper had been added. The heat was removed, and stirring was continued overnight. This reaction was repeated on the same scale. The two reaction mixtures were combined with water (140 mL), heated to boiling and filtered with suction. The tarry solid was boiled with water (100 mL) and filtered, and this process was repeated once. The three filtrates were combined and neutralized with aq. NH_4_OH (80 mL). The aqueous layer was extracted with CH_2_Cl_2_ (3 × 100 mL). The combined organic layers were washed with water (2 × 250 mL), dried over CaCl_2_ and concentrated in vacuo. The resulting solid was distilled under vacuum (0.1 torr), giving ~11 g of a 4:1 mixture of **3** and 1-naphthyl amine. This product was recrystallized from ethanol (85 mL) and water (15 mL), giving nearly pure **3** (5.2 g, 23.4 mmol, 35%). The analytical data were in accordance with those found in the literature [5].

### 3.3. 1-(8-Bromonaphthalen-1-yl)-1H-pyrrole *(**4**)*

Bromonaphthylamine **3** (0.50 g, 2.25 mmol) was combined with 2,5-dimethoxytetrahydrofuran (0.29 mL, 2.25 mmol) and acetic acid (10 mL), and the mixture was heated to reflux for 1 h. The reaction was cooled and added dropwise to ice-cold 1 M NaOH (200 mL). The aqueous layer was extracted with CH_2_Cl_2_ (2 × 50 mL). The combined organic layers were combined, dried over CaCl_2_ and concentrated in vacuo, giving 0.59 g of crude product. This material was distilled under vacuum (0.1 torr), giving mostly pure **4** (0.48 g, 78%) as a brown oil, which was used directly in the next step. NMR ^1^H (400 MHz, CDCl_3_): 7.91–7.81 (m, 3H), 7.47 (m, 2H), 7.28 (t, *J* = 7.8 Hz, 1H), 6.77 (m, 2H), 6.32 (m, 2H); ^13^C (400 MHz, CDCl_3_): *δ* 137.53, 136.42, 134.66, 130.05, 128.86, 128.81, 127.84, 126.63, 125.63, 125.18, 117.11, 108.92.

### 3.4. Ethyl 8-(1H-pyrrol-1-yl)-1-naphthoate *(**5**)*

Bromopyrrolylnaphthalene **4** (0.77 g, 2.8 mmol) was dissolved in THF (12 mL) and cooled to −78 °C under N_2_. A 2.7 M solution of *n*-BuLi in hexanes (1.26 mL, 3.4 mmol) was added slowly dropwise. The reaction was stirred at −78 °C for 30 min. Freshly distilled ethyl chloroformate (320 µL, 3.4 mmol) was added dropwise, and the reaction was allowed to warm to room temperature with continued stirring. The reaction was quenched with aq. NH_4_Cl (5 mL), diluted with aq. NH_4_Cl (100 mL) and extracted with CH_2_Cl_2_ (2 × 75 mL). The combined organic layers were dried over CaCl_2_ and concentrated in vacuo. Column chromatography of the residue on silica gel with gradient elution (0→17% ethyl acetate in hexanes) gave **5** (230 mg, 0.87 mmol, 31%) as a yellow oil. An earlier fraction (320 mg) was a 1:1 mixture with 1-pyrrolylnaphthalene. NMR ^1^H (400 MHz, CDCl_3_): 7.97 (d, *J* = 8.1 Hz, 1H), 7.86 (d, *J* = 8.1 Hz, 1H), 7.73 (d, *J* = 6.9 Hz, 1H), 7.56–7.46 (m, 3H), 6.91 (m, 2H), 6.32 (m, 2H), 3.88 (q, *J* = 7.2 Hz, 2H), 1.21, (t, *J* = 7.2 Hz, 3H); ^13^C (400 MHz, CDCl_3_): *δ* 168.33, 137.61, 135.41, 131.35, 130.43, 129.34, 128.29, 126.23, 126.17, 126.15, 125.4, 122.87, 109.58, 61.75, 14.07.

### 3.5. 7H-benzo[de]pyrrolo[1,2-a]quinolin-7-one *(**6**)*

Carboxyethylpyrrolylnaphthalene **5** (73 mg, 0.27 mmol) and p-toluenesulfonic acid monohydrate (80 mg, 0.42 mmol) were covered with toluene (18 mL). The mixture was heated at reflux for 2 h. After cooling, the mixture was diluted with CH_2_Cl_2_ (75 mL) and washed with 0.24 M aq. NaHCO_3_ (50 mL) and water (100 mL). The organic layer was dried over Na_2_SO_4_ and concentrated in vacuo. Column chromatography of the residue on silica gel with gradient elution (0→25% ethyl acetate in hexanes) gave **6** (42 mg, 0.19 mmol, 70%) as a yellow solid, m.p. 141–143 °C (lit. 145–146 °C) [6]. NMR ^1^H (400 MHz, CDCl_3_): 8.51 (d, *J* = 7.3 Hz, 1H), 8.01 (d, *J* = 8.2 Hz, 1H), 7.79–7.63 (m, 4H), 7.48 (dd, *J* = 7.9, 8.0 Hz, 1H), 7.33 (m, 1H), 6.54 (m, 1H); ^13^C (400 MHz, CDCl_3_): *δ* 174.66, 133.88, 133.45, 130.78, 130.70, 128.01, 127.74, 127.21, 126.40, 125.81, 122.28, 120.07, 116.34, 113.39, 112.28.

### 3.6. (8-(1H-pyrrol-1-yl)naphthalen-1-yl)(phenyl)methanol *(**7**)*

Bromopyrrolylnaphthalene **4** (280 mg, 1.0 mmol) was dissolved in THF (10 mL) and cooled to −78 °C under N_2_. A 2.5 M solution of *n*-BuLi in hexanes (0.44 mL, 1.1 mmol) was added slowly dropwise. The reaction was stirred at −78 °C for 30 min. Freshly distilled benzaldehyde (120 µL, 1.2 mmol) was added dropwise, and the reaction was allowed to warm to room temperature with continued stirring. The reaction was quenched with aq. NH_4_Cl (5 mL), diluted with aq. NH_4_Cl (100 mL) and extracted with CH_2_Cl_2_ (2 × 75 mL). The combined organic layers were dried over CaCl_2_ and concentrated in vacuo, giving 0.36 g of crude product. Column chromatography of the residue on silica gel with gradient elution (0→20% ethyl acetate in hexanes) gave **7** (210 mg, 0.70 mmol, 70%) as a brown amorphous solid., m.p. 105–107 °C. NMR ^1^H (400 MHz, CDCl_3_): 7.95 (d, *J* = 7.8 Hz, 1H), 7.87 (d, *J* = 8.0 Hz, 1H), 7.57 (d, *J* = 7.6 Hz, 1H), 7.50–7.42 (m, 3H), 7.23–7.13 (m, 3H), 7.08 (d, *J* = 6.9 Hz, 2H), 6.97 (m, 1H), 6.78 (m, 1H), 6.37 (m, 1H), 6.29 (m, 1H), 5.05 (s, 1H), 2.14 (br. s, 1H, OH); ^13^C (400 MHz, CDCl_3_): *δ* 145.1, 140.06, 137.26, 135.74, 130.52, 129.67, 129.21, 128.57, 128.27, 128.11, 127.02, 126.73, 126.32, 124.79, 123.96, 123.73, 109.95, 109.87, 70.41.

### 3.7. 7-Phenyl-10H-benzo[de]pyrrolo [1,2-a]quinolin-10-one *(**8**)*

Phenylmethanol **7** (0.26 g, 0.88 mmol) and pyridinium chlorochromate (PCC, 0.38 g, 1.8 mmol) were stirred overnight in CH_2_Cl_2_ (15 mL). TLC indicated an incomplete reaction. PCC (0.20 g, 0.93 mmol) was added, and the reaction was stirred overnight. PCC (0.05 g, 0.23 mmol) was added, and stirring continued for two days. The reaction was concentrated in vacuo. The residue was taken up in hexanes (50 mL). Celite (2 g) was added, and the mixture was stirred vigorously for 30 min. The slurry was filtered with suction, and the filtrate was concentrated in vacuo, giving 0.23 g of material. Column chromatography of the residue on silica gel with gradient elution (0→15% ethyl acetate in hexanes) gave **8** (110 mg, 0.37 mmol, 41%) as an orange red solid., m.p. 120–122 °C. NMR ^1^H (400 MHz, CDCl_3_): 8.89 (dd, *J* = 2.4, 6.5 Hz, 1H), 7.62 (d, *J* = 8.3 Hz, 1H), 7.54–7.46 (m, 5H), 7.43–7.39 (m, 2H), 7.29 (dd, *J* = 7.6, 8.0, 1H), 7.07 (d, *J* = 7.4 Hz, 1H), 7.02 (d, *J* = 5.8 Hz, 1H), 6.37 (d, *J* = 5.8 Hz, 1H); ^13^C (400 MHz, CDCl_3_): *δ* 168.03, 135.61, 134.11, 133.63, 133.52, 132.29, 130.59, 129.78, 128.94, 128.92, 128.22, 127.88, 127.26, 126.74, 124.27, 123.46, 123.30, 122.48, 111.26.

## 4. Conclusions

The preparation of 1,8-acyl pyrrolyl naphthalenes is challenging because of the tendency of the pyrrole moiety towards electrophilic substitution. The ester derivative **5** does not show much solvato-chromism due to the twisting of the pyrrole and the ester groups limiting their resonance interaction. In compound **6**, the carbonyl and pyrrole group are co-planar and have resonance interactions not only through the naphthalene but also through a direct connection. Its fluorescence is twice as solvato-chromic as that displayed by **5**. While **6** is weakly fluorescent in apolar, aprotic solvents, its emission is greatly enhanced in polar, protic solvents. Some of the enhancement is due to the lowering of the singlet state energy relative to the n→π* triplet state. However, the results suggest that the rate of ISC is also negatively affected by H-bonding in the excited state. H-bonding in the ground state is ruled out as the cause because of the small bathochromic shift in the absorption (less than 3 nm in ethanol) and the pronounced preferential solvation effect on the fluorescence with methanol and acetonitrile. Examples of protic solvents dramatically increasing the fluorescence intensity in aromatic carbonyls are seen with benzanthrone and thioxanthone [26,27,28,29]. With benzanthrone, H-bonding is thought to modify the spin-orbit coupling by affecting the out-of-plane vibrational modes and frequencies that serve to mix the in-plane n-orbital with the perpendicular π-orbitals [30]. For thioxanthone, the increase in fluorescence intensity is greater than two orders of magnitude going from benzene to methanol. Here, the effect of H-bonding is thought to primarily affect the relative energies of the π→π* singlet and n→π* triplet states. With **6**, H-bonding with protic media enhances the fluorescence in a way that correlates strongly with the H-bond donating solvent parameter SA. As such, **6** might be a sensitive sensor for H-bonding strength.

## Figures and Tables

**Table 1:** Multilinear regression results for the correlation of the relative integrated fluorescence intensity of **6** in various solvents vs. several combinations of SA, E_T_(30) and SdP solvent parameters.

	SA	E_T_(30)	SA	SdP	SA	E_T_(30)	SdP
coef.	1.177	0.014	1.598	0.093	0.917	0.025	−0.181
std error	0.132	0.004	0.090	0.065	0.153	0.005	0.071
log_10_*p*	−6.9	−3.0	−11.2	−0.8	−4.6	−3.6	−1.7
*F*-value	337		186		305		

**Table 2:** Non-linear least-square fitting values for Equation (7) for the preferential solvation plots in Figure 8. *Φ*_1_ is the quantum yield of the singly methanol-solvated species relative to the quantum yield in pure methanol. *Φ*_0_ is the quantum yield in the aprotic solvent.

RH	Toluene		Acetonitrile	
	*Γ*_1_	*Γ*_2_	*Γ*_1_	*Γ*_2_
*r*	0.65	0.66	0.26	0.26
*f*_1/0_	6.2	6.5	14.5	13.5
*f*_2/0_	1.6	1.6	29.2	29.2
*Φ*_1_	0.66	0.67	0.41	0.43
*Φ*_0_	0.03		0.20	

**Table 3:** Computed energies (kcal/mol) for transitions from the ground state (→) and the relaxed singlet excited state (←) with various solvent models.

	Vacuum		Toluene		Toluene Methanol *^c^*		Acetonitrile	
S_o→_S_1_ *^a^*	84.0		81.3		*^d^*		80.3	
S_o←_S_1_ *^a^*	56.9		53.4		51.9		49.2	
S_o→_T_nπ*_ *^b^*	74.2		80.9		*^d^*		80.2	
	HO-3→LU	0.63	HO-3→LU	0.42			HO-3→LU	0.42
	HO-3→LU+3	−0.25	HO-2→LU	0.34			HO-2→LU	0.37
							HO→LU+4	−0.23
S_o←_T_nπ*_ *^b^*	62.2		67.7		62.4		72.0	
	HO-3→LU	−0.47	HO-3→LU	0.65	HO-3→LU	0.49	HO-3→LU	0.65
	HO-1→LU	−0.29			HO→LU+3	−0.24		
	HO→LU+2	0.25						
S_1_-T_nπ*_ (→)	9.8		0.4		0.4		0.1	
S_1_-T_nπ*_ (←)	−5.3		−14.3		−10.5		−22.8	

*^a^* Singlet-to-singlet transitions are HO ↔ LU. *^b^* Singlet–triplet transitions show the constituent transitions and their coefficients for those with 11% or greater contribution. Only the triplet state with the largest HO-3 (n-π*) component is shown. All T_nπ*_ states are T_4_, except for the vacuum S_o→_T_nπ*_ entry, which is T_3_. *^c^* One methanol is H-bonded to **6**. *^d^* Energies assumed to be the same as those for toluene.

**Table 4:** Computed emission (em) and absorption (abs) maxima, dipole moments (em: S_1_, abs: S_0_), Stokes shifts and Mulliken charges on the carbonyl oxygen and pyrrole nitrogen for **6** and the related experimental values.

		Toluene			Acetonitrile	
		abs	em	em *^a^*	abs	em
calculated	λ_max_ (nm)	352	536	550	356	582
	Stokes shift (cm^−1^)		9800	10,200		10,900
	μ (D)	3.8	5.4	7.3	6.8	7.3
	O	−0.57	−0.60	−0.67	−0.62	−0.65
	N	0.12	0.16	0.17	0.11	0.16
exp.	λ_max_ (nm)	387	476	479	386	489
	Stokes shift (cm^−1^)		4800	5000		5400

*^a^* For the H-bonded complex with methanol. The experimental values for this complex are derived from the preferential solvation studies.

**Table 5:** Multilinear regression results for the correlation of the fluorescence center-of-gravity (ῦ_cog_) of **6** in various solvents vs. several combinations of the SA, E_T_(30) and SdP solvent parameters.

	E_T_(30)	SA	E_T_(30)	SA	E_T_(30)	SdP
coef.	−83	−3100	−42	−1700	−104	850
std error	8	300	13	500	8.0	230
log *p*	−7.7	−8.0	−2.1	−2.5	−8.8	−2.7
*F*-value	97	107	85		92	

## Data Availability

The data presented in this study are available either in this article or in its Supplementary Materials.

## Supplementary Materials

The following supporting information can be downloaded at https://www.mdpi.com/article/10.3390/molecules30071429/s1: crystallographic information files (CIF) for compounds **6**–**8**; checkCIF report from the Cambridge Crystallographic Data Centre (CCDC); Bond lengths [Å] and angles [°] for Compounds **6**–**8**; Crystal structures and refinement for Compounds **6**–**8**; Proton and carbon NMR spectra for **4**–**8**; Absorption maxima and molar absorptivities for **6** and **8**; Fluorescence maxima and intensities for **5**–**6** and **8**.
