# Supplementary material for: Peri-Substituted Acyl Pyrrolyl Naphthalenes: Synthesis, Reactions and Photophysical Properties
Source: Molecules. 2025 Mar 24;30(7):1429. doi: 10.3390/molecules30071429 (PMC11990698; doi:10.3390/molecules30071429)
Supplement: Supplementary file 1 [file molecules-30-01429-s001.zip › CheckCIF reports.pdf]

Compound 6

No syntax errors found.
Please wait while processing ....

CIF dictionary
Interpreting this report

Datablock: mo_CJA007_0m_a

Bond precision: C-C = 0.0042 A   Wavelength=0.71073

Cell:	a=30.5846(19)	b=4.7880(3)	c=14.5788(8)
	alpha=90	beta=108.541(2)	gamma=90

Temperature: 100 K

	Calculated	Reported
Volume	2024.1(2)	2024.1(2)
Space group	C 2	C 2
Hall group	C 2y	C 2y
Moiety formula	C15 H9 N O	C15 H9 N O
Sum formula	C15 H9 N O	C15 H9 N O
Mr	219.23	219.23
Dx,g cm-3	1.439	1.439
Z	8	8
Mu (mm-1)	0.091	0.091
F000	912.0	912.0
F000'	912.38	
h,k,lmax	37,5,18	37,5,18
Nref	3970[2236]	3959
Tmin,Tmax	0.988,0.998	0.819,0.982
Tmin'	0.963	

Correction method= # Reported T Limits: Tmin=0.819 Tmax=0.982
AbsCorr = NUMERICAL

Data completeness= 1.77/1.00   Theta(max)= 26.047

R(reflections)= 0.0467(3511)   wR2(reflections)= 0.1148(3959)

S = 1.027   Npar= 307

The following ALERTS were generated. Each ALERT has the format
test-name_ALERT_alert-type_alert-level.
Click on the hyperlinks for more details of the test.

Alert level C

```
STRVA01_ALERT_2_C           Chirality of atom sites is inverted?
           From the CIF: _refine_ls_abs_structure_Flack     0.800
           From the CIF: _refine_ls_abs_structure_Flack_su     1.000
PLAT089_ALERT_3_C Poor Data / Parameter Ratio (Zmax < 18) ........       7.27 Note
PLAT340_ALERT_3_C Low Bond Precision on  C-C Bonds ...............    0.00416 Ang.
PLAT907_ALERT_2_C Flack x > 0.5, Structure Needs to be Inverted? .       0.80 Check
```

Alert level G

```
PLAT032_ALERT_4_G Std. Uncertainty on Flack Parameter Value High .      1.000 Report
PLAT128_ALERT_4_G Alternate Setting for Input Space Group   C2             I2 Note
PLAT883_ALERT_1_G Absent Datum for _atom_sites_solution_primary ..     Please Do !
PLAT899_ALERT_4_G SHELXL2018 is Outdated    and Succeeded by SHELXL     2019/3 Note
PLAT910_ALERT_3_G Missing # of FCF Reflection(s) Below Theta(Min).          3 Note
               2  0  0,   -2  0  1,    0  0  1,
PLAT916_ALERT_2_G Hooft y and Flack x Parameter Values Differ by .       0.30 Check
PLAT969_ALERT_5_G The 'Henn et al.' R-Factor-gap value ...........      1.496 Note
              Predicted wR2: Based on SigI**2  7.67 or SHELX Weight 11.18
PLAT978_ALERT_2_G Number C-C Bonds with Positive Residual Density.          0 Info
```

0 ALERT level A = Most likely a serious problem - resolve or explain
0 ALERT level B = A potentially serious problem, consider carefully
4 ALERT level C = Check. Ensure it is not caused by an omission or oversight
8 ALERT level G = General information/check it is not something unexpected

1 ALERT type 1 CIF construction/syntax error, inconsistent or missing data
4 ALERT type 2 Indicator that the structure model may be wrong or deficient
3 ALERT type 3 Indicator that the structure quality may be low

```
    3 ALERT type 4 Improvement, methodology, query or suggestion
    1 ALERT type 5 Informative message, check
```

It is advisable to attempt to resolve as many as possible of the alerts in all categories. Often the minor alerts point to easily fixed oversights, errors and omissions in your CIF or refinement strategy, so attention to these fine details can be worthwhile. In order to resolve some of the more serious problems it may be necessary to carry out additional measurements or structure refinements. However, the purpose of your study may justify the reported deviations and the more serious of these should normally be commented upon in the discussion or experimental section of a paper or in the "special_details" fields of the CIF. checkCIF was carefully designed to identify outliers and unusual parameters, but every test has its limitations and alerts that are not important in a particular case may appear. Conversely, the absence of alerts does not guarantee there are no aspects of the results needing attention. It is up to the individual to critically assess their own results and, if necessary, seek expert advice.

Publication of your CIF in IUCr journals

A basic structural check has been run on your CIF. These basic checks will be run on all CIFs submitted for publication in IUCr journals (*Acta Crystallographica*, *Journal of Applied Crystallography*, *Journal of Synchrotron Radiation*); however, if you intend to submit to *Acta Crystallographica Section C* or *E* or *IUCrData*, you should make sure that full publication checks are run on the final version of your CIF prior to submission.

Publication of your CIF in other journals

Please refer to the *Notes for Authors* of the relevant journal for any special instructions relating to CIF submission.

PLATON version of 02/02/2025; check.def file version of 02/02/2025

Datablock mo_CJA007_0m_a - ellipsoid plot

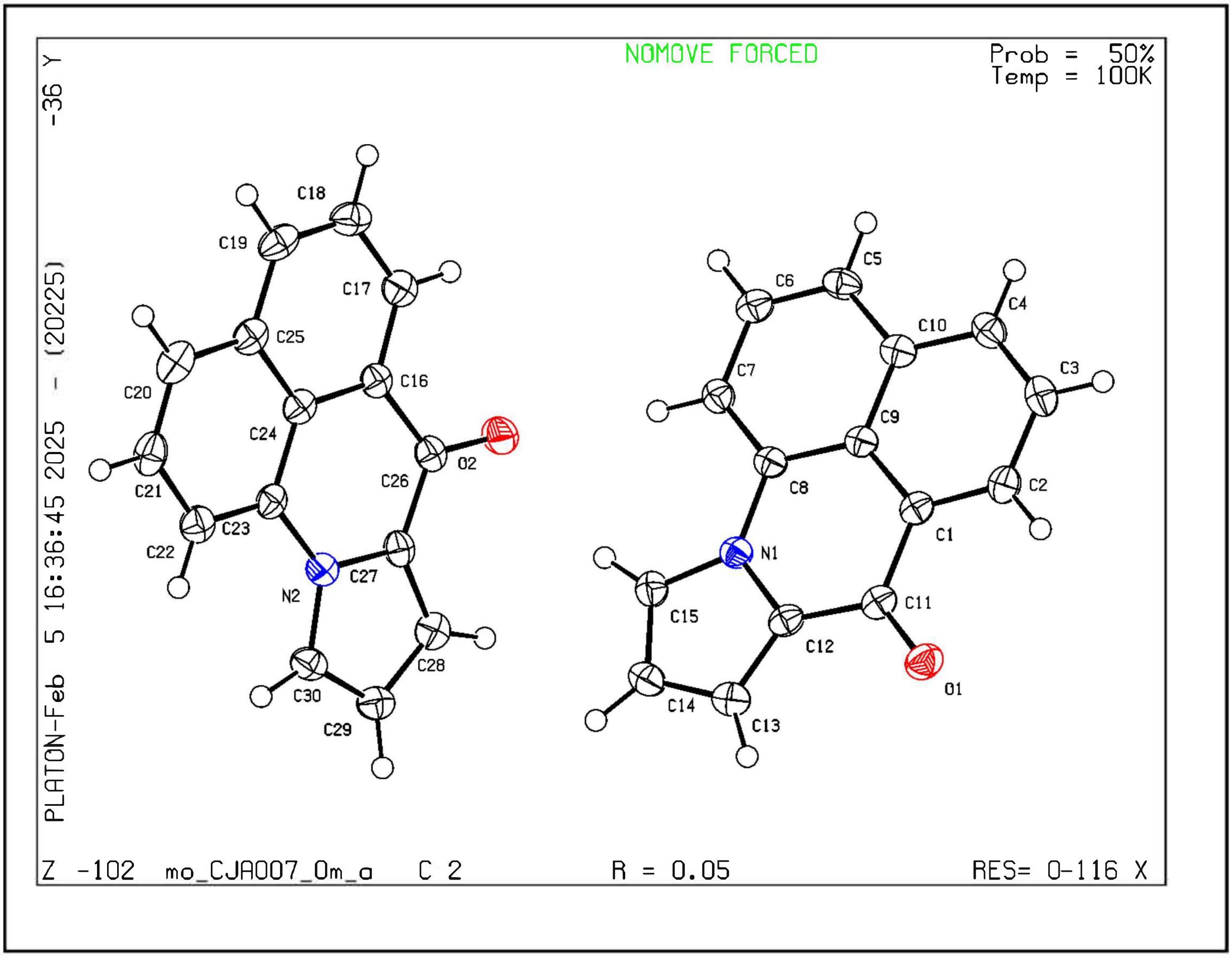

[Download CIF editor (publCIF) from the IUCr](#)
[Download CIF editor (enCIFer) from the CCDC](#)
[Test a new CIF entry](#)

Compound 7

No syntax errors found.
Please wait while processing

CIF dictionary
Interpreting this report

Datablock: mo_CJA006_0m_a

Bond precision:	C-C = 0.0020 A		Wavelength=0.71073
Cell:	a=8.5180(8)	b=10.0713(11)	c=10.1653(11)
	alpha=62.330(4)	beta=80.923(4)	gamma=85.320(4)
Temperature:	100 K		

	Calculated	Reported
Volume	762.62(14)	762.62(14)
Space group	P -1	P -1
Hall group	-P 1	-P 1
Moiety formula	C21 H17 N O	C21 H17 N O
Sum formula	C21 H17 N O	C21 H17 N O
Mr	299.36	299.36
Dx,g cm-3	1.304	1.304
Z	2	2
Mu (mm-1)	0.080	0.080
F000	316.0	316.0
F000'	316.12	
h,k,lmax	10,12,12	10,12,12
Nref	3006	3004
Tmin,Tmax	0.995,0.998	0.935,0.986
Tmin'	0.992	

Correction method= # Reported T Limits: Tmin=0.935 Tmax=0.986
AbsCorr = NUMERICAL

Data completeness= 0.999 Theta(max)= 26.013

R(reflections)= 0.0407(2492) wR2(reflections)= 0.1454(3004)

S = 1.145 Npar= 208

The following ALERTS were generated. Each ALERT has the format
test-name_ALERT_alert-type_alert-level.
Click on the hyperlinks for more details of the test.

Alert level C

```
PLAT085_ALERT_2_C SHELXL Default Weighting Scheme is not Optimized     Please Check
PLAT417_ALERT_2_C Short Inter D-H..H-D        H1        ..H1             2.11 Ang.
                                          1-x,1-y,1-z           2_666 Check
PLAT911_ALERT_3_C Missing FCF Refl Between Thmin & STh/L=    0.600           2 Report
                1   0  0,   -4  1  0,
```

Alert level G

```
PLAT007_ALERT_5_G Number of Unrefined Donor-H Atoms                          1 Report
              H1
PLAT154_ALERT_1_G The s.u.'s on the Cell Angles are Equal ..(Note)      0.004 Degree
PLAT180_ALERT_4_G Check Cell Rounding: # of Values Ending with 0 =          3 Note
PLAT793_ALERT_4_G Model has Chirality at C1'              (Centro SpGr)      S Verify
PLAT883_ALERT_1_G Absent Datum for _atom_sites_solution_primary ..     Please Do !
PLAT899_ALERT_4_G SHELXL2018 is Outdated    and Succeeded by SHELXL     2019/3 Note
PLAT965_ALERT_2_G The SHELXL WEIGHT Optimisation has not Converged     Please Check
PLAT969_ALERT_5_G The 'Henn et al.' R-Factor-gap value ...........      4.162 Note
              Predicted wR2: Based on SigI**2   3.49 or SHELX Weight 12.69
PLAT978_ALERT_2_G Number C-C Bonds with Positive Residual Density.          2 Info
```

0 ALERT level A Most likely a serious problem - resolve or explain
0 ALERT level B A potentially serious problem, consider carefully
3 ALERT level C Check. Ensure it is not caused by an omission or oversight
9 ALERT level G General information/check it is not something unexpected

2 ALERT type 1 CIF construction/syntax error, inconsistent or missing data
4 ALERT type 2 Indicator that the structure model may be wrong or deficient
1 ALERT type 3 Indicator that the structure quality may be low

```
3 ALERT type 4 Improvement, methodology, query or suggestion
2 ALERT type 5 Informative message, check
```

It is advisable to attempt to resolve as many as possible of the alerts in all categories. Often the minor alerts point to easily fixed oversights, errors and omissions in your CIF or refinement strategy, so attention to these fine details can be worthwhile. In order to resolve some of the more serious problems it may be necessary to carry out additional measurements or structure refinements. However, the purpose of your study may justify the reported deviations and the more serious of these should normally be commented upon in the discussion or experimental section of a paper or in the "special_details" fields of the CIF. checkCIF was carefully designed to identify outliers and unusual parameters, but every test has its limitations and alerts that are not important in a particular case may appear. Conversely, the absence of alerts does not guarantee there are no aspects of the results needing attention. It is up to the individual to critically assess their own results and, if necessary, seek expert advice.

Publication of your CIF in IUCr journals

A basic structural check has been run on your CIF. These basic checks will be run on all CIFs submitted for publication in IUCr journals (*Acta Crystallographica*, *Journal of Applied Crystallography*, *Journal of Synchrotron Radiation*); however, if you intend to submit to *Acta Crystallographica Section C* or *E* or *IUCrData*, you should make sure that full publication checks are run on the final version of your CIF prior to submission.

Publication of your CIF in other journals

Please refer to the *Notes for Authors* of the relevant journal for any special instructions relating to CIF submission.

PLATON version of 02/02/2025; check.def file version of 02/02/2025

Datablock mo_CJA006_0m_a - ellipsoid plot

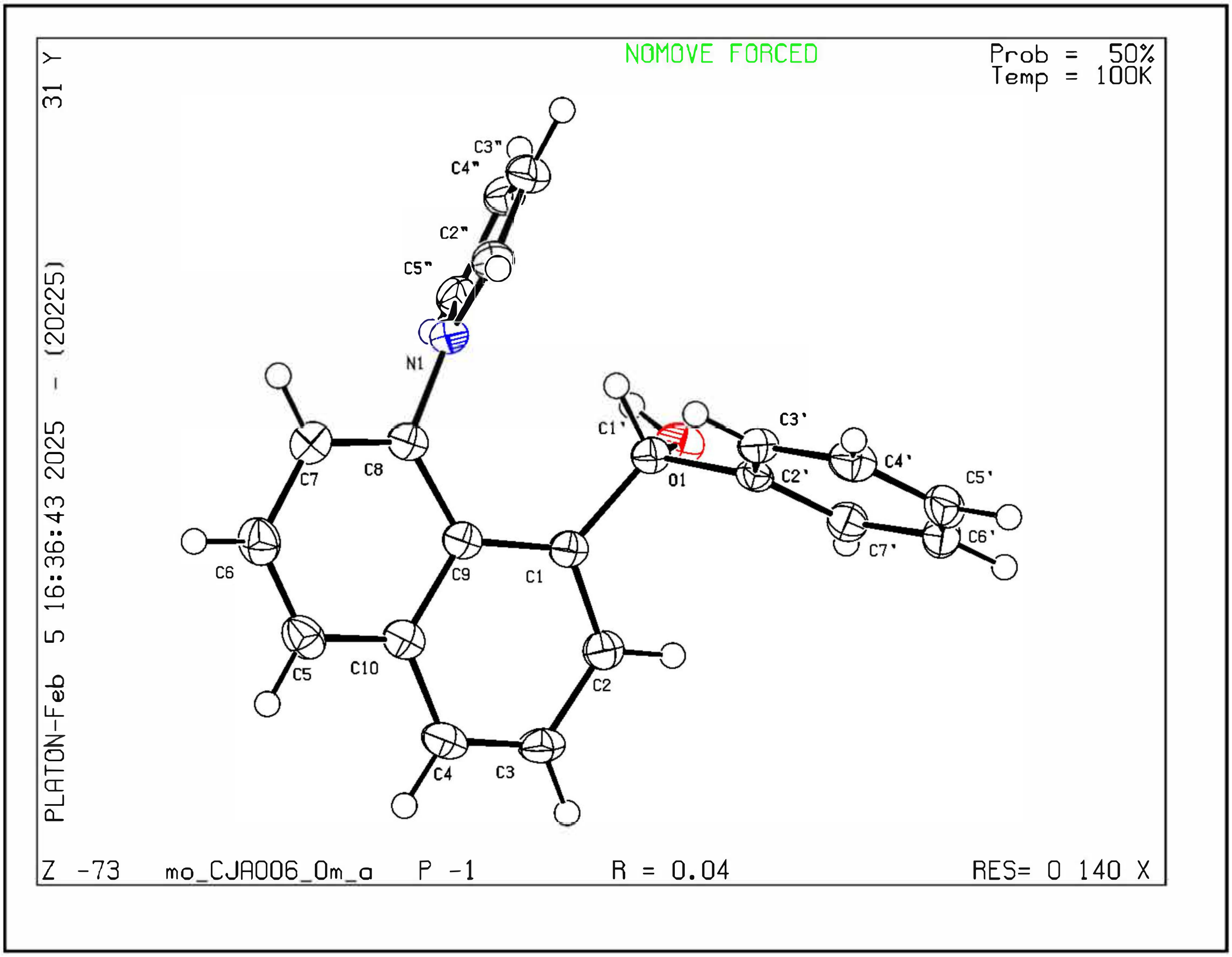

[Download CIF editor (publCIF) from the IUCr]
[Download CIF editor (enCIFer) from the CCDC]
[Test a new CIF entry]

Compound 8

No syntax errors found.
Please wait while processing ....

CIF dictionary
Interpreting this report

Datablock: mo_CJA008_0m_a

Bond precision: C-C = 0.0020 A Wavelength=0.71073

Cell: a=23.606(2) b=9.9979(9) c=13.7241(13)
alpha=90 beta=118.225(3) gamma=90

Temperature: 100 K

	Calculated	Reported
Volume	2853.9(5)	2853.9(5)
Space group	C 2/c	C 2/c
Hall group	-C 2yc	-C 2yc
Moiety formula	C21 H13 N O	C21 H13 N O
Sum formula	C21 H13 N O	C21 H13 N O
Mr	295.32	295.32
Dx,g cm-3	1.375	1.375
Z	8	8
Mu (mm-1)	0.085	0.085
F000	1232.0	1232.0
F000'	1232.50	
h,k,lmax	29,12,16	29,12,16
Nref	2812	2810
Tmin,Tmax	0.971,0.980	0.646,0.704
Tmin'	0.939	

Correction method= # Reported T Limits: Tmin=0.646 Tmax=0.704
AbsCorr = MULTI-SCAN

Data completeness= 0.999 Theta(max)= 26.019

R(reflections)= 0.0441(2467) wR2(reflections)= 0.1257(2810)

S = 1.070 Npar= 209

The following ALERTS were generated. Each ALERT has the format
test-name_ALERT_alert-type_alert-level.
Click on the hyperlinks for more details of the test.

Alert level B

```
PLAT919_ALERT_3_B Reflection # Likely Affected by the Beamstop ...          1 Check
                 2   0   0,
```

Alert level C

```
PLAT911_ALERT_3_C Missing FCF Refl Between Thmin & STh/L=    0.600          2 Report
                 0   8   0,   -8   0   2,
PLAT934_ALERT_3_C Number of (Iobs-Icalc)/Sigma(W) > 10 Outliers ..          1 Check
                 2   0   0,
```

Alert level G

```
PLAT063_ALERT_4_G Crystal Size Possibly too Large for Beam Size ..       0.74 mm
PLAT128_ALERT_4_G Alternate Setting for Input Space Group     C2/c       I2/a Note
PLAT883_ALERT_1_G Absent Datum for _atom_sites_solution_primary ..     Please Do !
PLAT899_ALERT_4_G SHELXL2018 is Outdated    and Succeeded by SHELXL     2019/3 Note
PLAT969_ALERT_5_G The 'Henn et al.' R-Factor-gap value ...........      3.126 Note
               Predicted wR2: Based on SigI**2  4.02 or SHELX Weight 11.75
PLAT978_ALERT_2_G Number C-C Bonds with Positive Residual Density.          7 Info
```

```
0 ALERT level A = Most likely a serious problem - resolve or explain
1 ALERT level B = A potentially serious problem, consider carefully
2 ALERT level C = Check. Ensure it is not caused by an omission or oversight
6 ALERT level G = General information/check it is not something unexpected

1 ALERT type 1 CIF construction/syntax error, inconsistent or missing data
1 ALERT type 2 Indicator that the structure model may be wrong or deficient
3 ALERT type 3 Indicator that the structure quality may be low
```

```
  3 ALERT type 4 Improvement, methodology, query or suggestion
  1 ALERT type 5 Informative message, check
```

It is advisable to attempt to resolve as many as possible of the alerts in all categories. Often the minor alerts point to easily fixed oversights, errors and omissions in your CIF or refinement strategy, so attention to these fine details can be worthwhile. In order to resolve some of the more serious problems it may be necessary to carry out additional measurements or structure refinements. However, the purpose of your study may justify the reported deviations and the more serious of these should normally be commented upon in the discussion or experimental section of a paper or in the "special_details" fields of the CIF. checkCIF was carefully designed to identify outliers and unusual parameters, but every test has its limitations and alerts that are not important in a particular case may appear. Conversely, the absence of alerts does not guarantee there are no aspects of the results needing attention. It is up to the individual to critically assess their own results and, if necessary, seek expert advice.

Publication of your CIF in IUCr journals

A basic structural check has been run on your CIF. These basic checks will be run on all CIFs submitted for publication in IUCr journals (*Acta Crystallographica*, *Journal of Applied Crystallography*, *Journal of Synchrotron Radiation*); however, if you intend to submit to *Acta Crystallographica Section C* or *E* or *IUCrData*, you should make sure that full publication checks are run on the final version of your CIF prior to submission.

Publication of your CIF in other journals

Please refer to the *Notes for Authors* of the relevant journal for any special instructions relating to CIF submission.

PLATON version of 02/02/2025; check.def file version of 02/02/2025

Datablock mo_CJA008_0m_a - ellipsoid plot

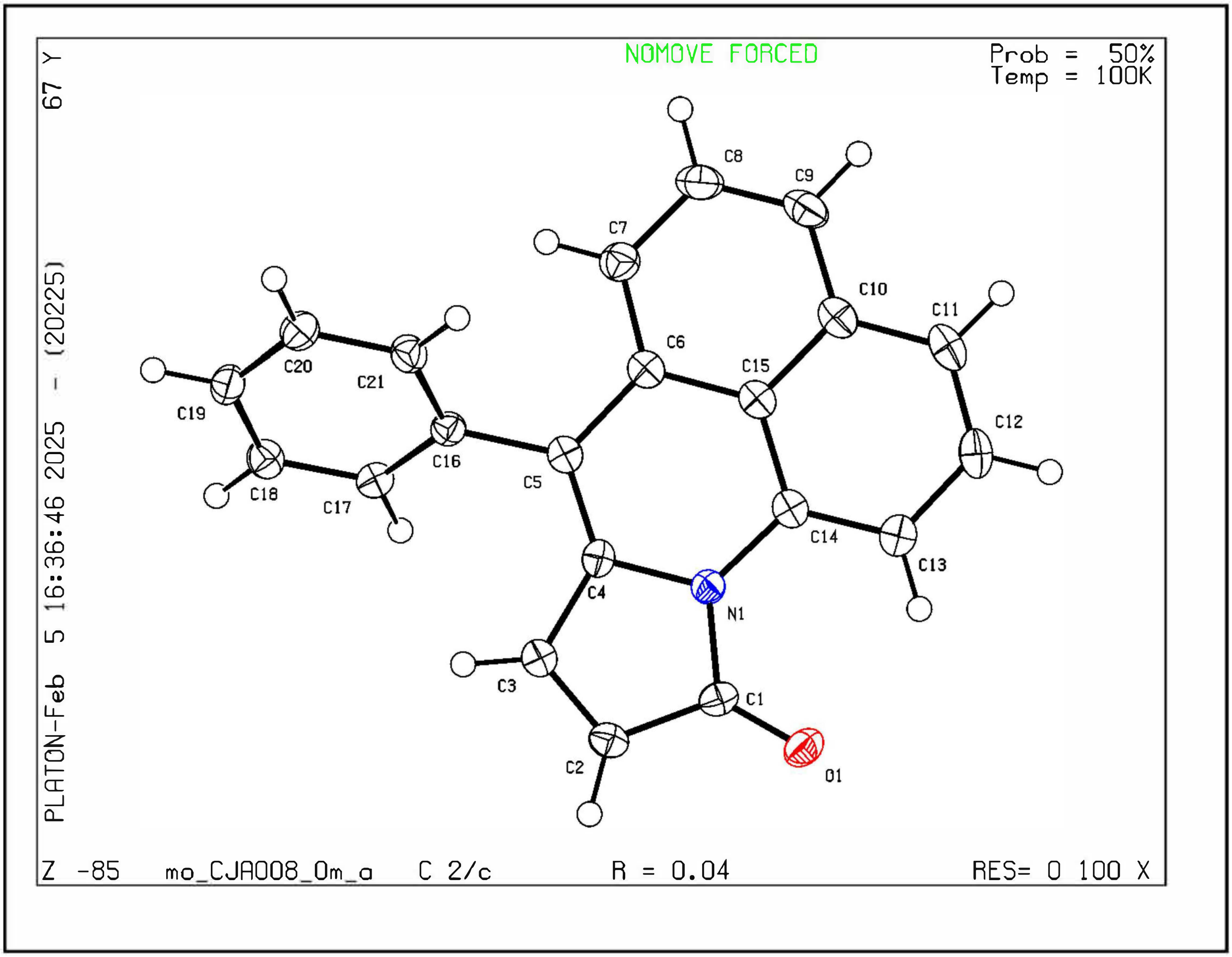

Download CIF editor (publCIF) from the IUCr
Download CIF editor (enCIFer) from the CCDC
Test a new CIF entry
