# Supplementary material for: Peri-Substituted Acyl Pyrrolyl Naphthalenes: Synthesis, Reactions and Photophysical Properties
Source: Molecules. 2025 Mar 24;30(7):1429. doi: 10.3390/molecules30071429 (PMC11990698; doi:10.3390/molecules30071429)
Supplement: Supplementary file 1 [file molecules-30-01429-s001.zip › supplementary information.pdf]

Peri-Substituted Acyl Pyrrolyl Naphthalenes: Synthesis, Reactions and Photophysical Properties

Junkai Zhao, Robert Pike, and Christopher Abelt*

College of William and Mary, Department of Chemistry, Williamsburg, Virginia, 23185, USA

Supplementary Information

Table of Contents

Title and Table of Contents S1

X-ray crystal structure data........ S2

Bond lengths [Å] and angles [°] for Compound **6** S2

Bond lengths [Å] and angles [°] for Compound **7** S4

Bond lengths [Å] and angles [°] for Compound **8** S6

Crystal data and structure refinement for Compound **6**........ S8

Crystal data and structure refinement for Compound **7**........ S9

Crystal data and structure refinement for Compound **8**........ S10

NMR spectroscopy S11

Figure S1. ^{1}H NMR spectrum of **4**........ S11

Figure S2. ^{13}CNMR spectrum of **4** S12

Figure S3. ^{1}H NMR spectrum of **5**........ S13

Figure S4. ^{13}C NMR spectrum of **5** S14

Figure S5. ^{1}H NMR spectrum of **6**........ S15

Figure S6. ^{13}C NMR spectrum of **6** S16

Figure S7. ^{1}H NMR spectrum of **7**........ S17

Figure S8. ^{13}CNMR spectrum of **7** S18

Figure S9. ^{1}H NMR spectrum of **8**........ S19

Figure S10. ^{13}CNMR spectrum of **8** S20

Absorption and fluorescence spectroscopy S21

Table S1. Absorption maxima and molar absorptivities for **6** and **8** S21

Table S2. Fluorescence maxima and intensities for **5**-**6** and **8** S21

Bond lengths [Å] and angles [°] for Compound **6**.

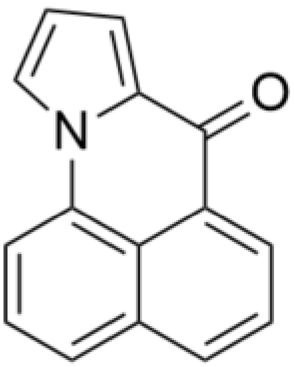

6

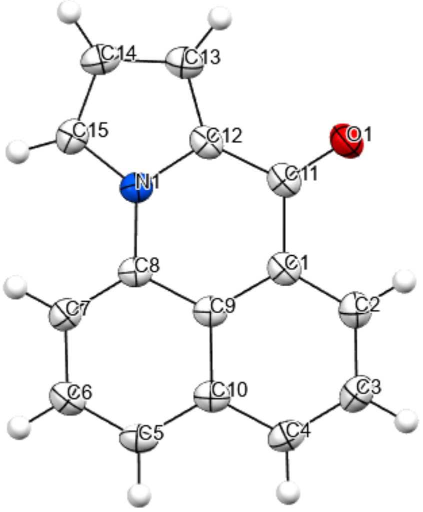

O(1)-C(11)	1.234(3)
N(1)-C(15)	1.371(4)
N(1)-C(12)	1.395(4)
N(1)-C(8)	1.412(4)
C(1)-C(2)	1.372(4)
C(1)-C(9)	1.426(4)
C(1)-C(11)	1.482(4)
C(2)-C(3)	1.400(4)
C(2)-H(2)	0.9500
C(3)-C(4)	1.369(4)
C(3)-H(3)	0.9500
C(4)-C(10)	1.411(4)
C(4)-H(4)	0.9500
C(5)-C(6)	1.366(4)
C(5)-C(10)	1.416(4)
C(5)-H(5)	0.9500
C(6)-C(7)	1.405(4)
C(6)-H(6)	0.9500
C(7)-C(8)	1.372(4)
C(7)-H(7)	0.9500
C(8)-C(9)	1.418(4)
C(9)-C(10)	1.423(4)
C(11)-C(12)	1.444(4)
C(12)-C(13)	1.383(4)
C(13)-C(14)	1.403(5)
C(13)-H(13)	0.9500
C(14)-C(15)	1.376(4)
C(14)-H(14)	0.9500
C(15)-H(15)	0.9500

C(15)-N(1)-C(12)	108.7(2)
C(15)-N(1)-C(8)	127.8(2)
C(12)-N(1)-C(8)	123.5(2)
C(2)-C(1)-C(9)	119.9(3)
C(2)-C(1)-C(11)	120.3(3)
C(9)-C(1)-C(11)	119.9(3)
C(1)-C(2)-C(3)	121.2(3)
C(1)-C(2)-H(2)	119.4
C(3)-C(2)-H(2)	119.4
C(4)-C(3)-C(2)	120.0(3)
C(4)-C(3)-H(3)	120.0
C(2)-C(3)-H(3)	120.0
C(3)-C(4)-C(10)	121.1(3)
C(3)-C(4)-H(4)	119.4
C(10)-C(4)-H(4)	119.4
C(6)-C(5)-C(10)	120.5(3)

C(6)-C(5)-H(5)	119.7	C(13)-C(12)-N(1)	107.4(3)
C(10)-C(5)-H(5)	119.7	C(13)-C(12)-C(11)	131.3(3)
C(5)-C(6)-C(7)	120.9(3)	N(1)-C(12)-C(11)	121.3(3)
C(5)-C(6)-H(6)	119.5	C(12)-C(13)-C(14)	107.7(3)
C(7)-C(6)-H(6)	119.5	C(12)-C(13)-H(13)	126.2
C(8)-C(7)-C(6)	119.9(3)	C(14)-C(13)-H(13)	126.2
C(8)-C(7)-H(7)	120.1	C(15)-C(14)-C(13)	108.0(3)
C(6)-C(7)-H(7)	120.1	C(15)-C(14)-H(14)	126.0
C(7)-C(8)-N(1)	122.1(3)	C(13)-C(14)-H(14)	126.0
C(7)-C(8)-C(9)	121.0(2)	N(1)-C(15)-C(14)	108.2(3)
N(1)-C(8)-C(9)	117.0(2)	N(1)-C(15)-H(15)	125.9
C(8)-C(9)-C(10)	118.7(2)	C(14)-C(15)-H(15)	125.9
C(8)-C(9)-C(1)	122.3(2)	C(27)-C(28)-H(28)	126.3
C(10)-C(9)-C(1)	118.9(3)	C(29)-C(28)-H(28)	126.3
C(4)-C(10)-C(5)	122.1(3)	C(30)-C(29)-C(28)	107.8(3)
C(4)-C(10)-C(9)	118.9(3)	C(30)-C(29)-H(29)	126.1
C(5)-C(10)-C(9)	119.0(3)	C(28)-C(29)-H(29)	126.1
O(1)-C(11)-C(12)	122.0(3)	C(29)-C(30)-N(2)	108.6(3)
O(1)-C(11)-C(1)	122.0(3)	C(29)-C(30)-H(30)	125.7
C(12)-C(11)-C(1)	116.0(2)	N(2)-C(30)-H(30)	125.7

Bond lengths [Å] and angles [°] for Compound **7**.

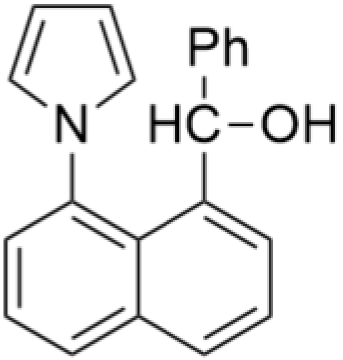

7

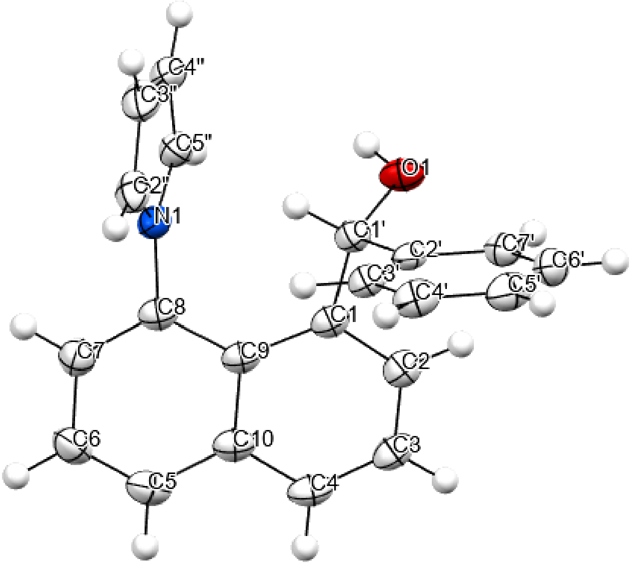

O(1)-C(1')	1.4418(15)	C(1')-C(2')	1.5181(17)
O(1)-H(1)	0.8400	C(1')-H(1')	1.0000
N(1)-C(5")	1.3718(17)	C(2')-C(3')	1.3904(17)
N(1)-C(2")	1.3739(17)	C(2')-C(7')	1.3907(19)
N(1)-C(8)	1.4289(16)	C(3')-C(4')	1.3908(19)
C(1)-C(2)	1.3780(19)	C(3')-H(3')	0.9500
C(1)-C(9)	1.4406(18)	C(4')-C(5')	1.382(2)
C(1)-C(1')	1.5368(17)	C(4')-H(4')	0.9500
C(2)-C(3)	1.4019(18)	C(5')-C(6')	1.381(2)
C(2)-H(2)	0.9500	C(5')-H(5')	0.9500
C(3)-C(4)	1.364(2)	C(6')-C(7')	1.3867(19)
C(3)-H(3)	0.9500	C(6')-H(6')	0.9500
C(4)-C(10)	1.411(2)	C(7')-H(7')	0.9500
C(4)-H(4)	0.9500	C(2")-C(3")	1.364(2)
C(5)-C(6)	1.360(2)	C(2")-H(2")	0.9500
C(5)-C(10)	1.4239(19)	C(3")-C(4")	1.410(2)
C(5)-H(5)	0.9500	C(3")-H(3")	0.9500
C(6)-C(7)	1.4036(19)	C(4")-C(5")	1.3664(19)
C(6)-H(6)	0.9500	C(4")-H(4")	0.9500
C(7)-C(8)	1.3712(18)	C(5")-H(5")	0.9500
C(7)-H(7)	0.9500		
C(8)-C(9)	1.4320(19)	C(1')-O(1)-H(1)	109.5
C(9)-C(10)	1.4352(18)	C(5")-N(1)-C(2")	108.78(12)

C(5")-N(1)-C(8)	125.50(11)	C(2')-C(1')-C(1)	111.62(10)
C(2")-N(1)-C(8)	125.65(11)	O(1)-C(1')-H(1')	109.1
C(2)-C(1)-C(9)	118.63(12)	C(2')-C(1')-H(1')	109.1
C(2)-C(1)-C(1')	115.77(11)	C(1)-C(1')-H(1')	109.1
C(9)-C(1)-C(1')	125.54(11)	C(3')-C(2')-C(7')	118.62(12)
C(1)-C(2)-C(3)	122.88(12)	C(3')-C(2')-C(1')	118.83(11)
C(1)-C(2)-H(2)	118.6	C(7')-C(2')-C(1')	122.53(11)
C(3)-C(2)-H(2)	118.6	C(2')-C(3')-C(4')	120.60(13)
C(4)-C(3)-C(2)	119.53(12)	C(2')-C(3')-H(3')	119.7
C(4)-C(3)-H(3)	120.2	C(4')-C(3')-H(3')	119.7
C(2)-C(3)-H(3)	120.2	C(5')-C(4')-C(3')	120.34(12)
C(3)-C(4)-C(10)	120.43(12)	C(5')-C(4')-H(4')	119.8
C(3)-C(4)-H(4)	119.8	C(3')-C(4')-H(4')	119.8
C(10)-C(4)-H(4)	119.8	C(4')-C(5')-C(6')	119.31(12)
C(6)-C(5)-C(10)	121.32(13)	C(4')-C(5')-H(5')	120.3
C(6)-C(5)-H(5)	119.3	C(6')-C(5')-H(5')	120.3
C(10)-C(5)-H(5)	119.3	C(5')-C(6')-C(7')	120.64(13)
C(5)-C(6)-C(7)	119.19(12)	C(5')-C(6')-H(6')	119.7
C(5)-C(6)-H(6)	120.4	C(7')-C(6')-H(6')	119.7
C(7)-C(6)-H(6)	120.4	C(6')-C(7')-C(2')	120.48(12)
C(8)-C(7)-C(6)	121.18(13)	C(6')-C(7')-H(7')	119.8
C(8)-C(7)-H(7)	119.4	C(2')-C(7')-H(7')	119.8
C(6)-C(7)-H(7)	119.4	C(3")-C(2")-N(1)	108.10(12)
C(7)-C(8)-N(1)	116.20(12)	C(3")-C(2")-H(2")	126.0
C(7)-C(8)-C(9)	121.98(12)	N(1)-C(2")-H(2")	126.0
N(1)-C(8)-C(9)	121.77(11)	C(2")-C(3")-C(4")	107.56(12)
C(8)-C(9)-C(10)	115.65(12)	C(2")-C(3")-H(3")	126.2
C(8)-C(9)-C(1)	126.71(12)	C(4")-C(3")-H(3")	126.2
C(10)-C(9)-C(1)	117.64(12)	C(5")-C(4")-C(3")	107.49(12)
C(4)-C(10)-C(5)	119.22(12)	C(5")-C(4")-H(4")	126.3
C(4)-C(10)-C(9)	120.48(12)	C(3")-C(4")-H(4")	126.3
C(5)-C(10)-C(9)	120.29(12)	C(4")-C(5")-N(1)	108.07(12)
O(1)-C(1')-C(2')	108.21(10)	C(4")-C(5")-H(5")	126.0
O(1)-C(1')-C(1)	109.74(10)	N(1)-C(5")-H(5")	126.0

—

Bond lengths [Å] and angles [°] for Compound **8**.

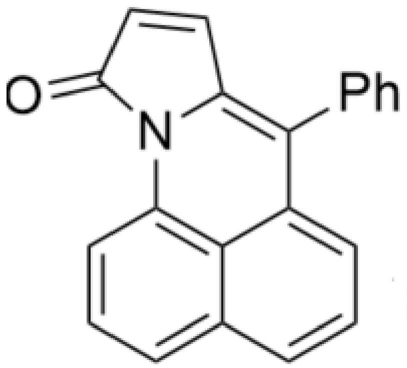

8

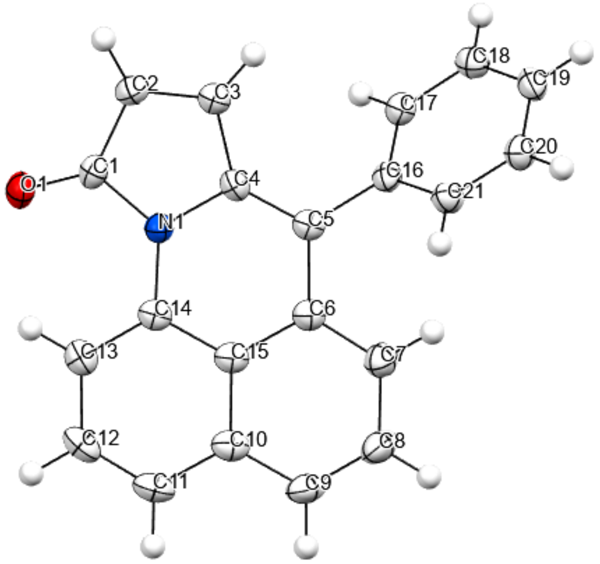

O(1)-C(1)	1.2234(16)	C(12)-C(13)	1.407(2)
N(1)-C(1)	1.4035(17)	C(12)-H(12)	0.9500
N(1)-C(4)	1.4102(16)	C(13)-C(14)	1.3826(18)
N(1)-C(14)	1.4113(17)	C(13)-H(13)	0.9500
C(1)-C(2)	1.4616(19)	C(14)-C(15)	1.4219(19)
C(2)-C(3)	1.3404(19)	C(16)-C(17)	1.3948(19)
C(2)-H(2)	0.9500	C(16)-C(21)	1.4025(19)
C(3)-C(4)	1.4458(19)	C(17)-C(18)	1.3908(18)
C(3)-H(3)	0.9500	C(17)-H(17)	0.9500
C(4)-C(5)	1.3594(19)	C(18)-C(19)	1.3831(19)
C(5)-C(6)	1.4637(18)	C(18)-H(18)	0.9500
C(5)-C(16)	1.4882(17)	C(19)-C(20)	1.389(2)
C(6)-C(7)	1.3859(19)	C(19)-H(19)	0.9500
C(6)-C(15)	1.4349(18)	C(20)-C(21)	1.3868(19)
C(7)-C(8)	1.404(2)	C(20)-H(20)	0.9500
C(7)-H(7)	0.9500	C(21)-H(21)	0.9500
C(8)-C(9)	1.368(2)		
C(8)-H(8)	0.9500	C(1)-N(1)-C(4)	109.77(10)
C(9)-C(10)	1.415(2)	C(1)-N(1)-C(14)	128.05(11)
C(9)-H(9)	0.9500	C(4)-N(1)-C(14)	121.92(11)
C(10)-C(11)	1.4154(19)	O(1)-C(1)-N(1)	125.21(12)
C(10)-C(15)	1.4255(19)	O(1)-C(1)-C(2)	129.25(12)
C(11)-C(12)	1.363(2)	N(1)-C(1)-C(2)	105.52(11)
C(11)-H(11)	0.9500	C(3)-C(2)-C(1)	109.32(12)

C(3)-C(2)-H(2)	125.3	C(11)-C(12)-H(12)	119.3
C(1)-C(2)-H(2)	125.3	C(13)-C(12)-H(12)	119.3
C(2)-C(3)-C(4)	109.29(12)	C(14)-C(13)-C(12)	119.44(13)
C(2)-C(3)-H(3)	125.4	C(14)-C(13)-H(13)	120.3
C(4)-C(3)-H(3)	125.4	C(12)-C(13)-H(13)	120.3
C(5)-C(4)-N(1)	122.31(12)	C(13)-C(14)-N(1)	122.42(12)
C(5)-C(4)-C(3)	131.58(12)	C(13)-C(14)-C(15)	120.99(12)
N(1)-C(4)-C(3)	106.09(11)	N(1)-C(14)-C(15)	116.59(11)
C(4)-C(5)-C(6)	118.61(12)	C(14)-C(15)-C(10)	118.38(12)
C(4)-C(5)-C(16)	120.04(11)	C(14)-C(15)-C(6)	122.06(12)
C(6)-C(5)-C(16)	121.34(11)	C(10)-C(15)-C(6)	119.56(12)
C(7)-C(6)-C(15)	119.11(12)	C(17)-C(16)-C(21)	118.36(12)
C(7)-C(6)-C(5)	122.66(12)	C(17)-C(16)-C(5)	121.23(11)
C(15)-C(6)-C(5)	118.23(11)	C(21)-C(16)-C(5)	120.37(12)
C(6)-C(7)-C(8)	120.86(13)	C(18)-C(17)-C(16)	120.59(12)
C(6)-C(7)-H(7)	119.6	C(18)-C(17)-H(17)	119.7
C(8)-C(7)-H(7)	119.6	C(16)-C(17)-H(17)	119.7
C(9)-C(8)-C(7)	120.68(13)	C(19)-C(18)-C(17)	120.40(12)
C(9)-C(8)-H(8)	119.7	C(19)-C(18)-H(18)	119.8
C(7)-C(8)-H(8)	119.7	C(17)-C(18)-H(18)	119.8
C(8)-C(9)-C(10)	121.01(13)	C(18)-C(19)-C(20)	119.76(12)
C(8)-C(9)-H(9)	119.5	C(18)-C(19)-H(19)	120.1
C(10)-C(9)-H(9)	119.5	C(20)-C(19)-H(19)	120.1
C(9)-C(10)-C(11)	121.95(13)	C(21)-C(20)-C(19)	120.02(13)
C(9)-C(10)-C(15)	118.68(13)	C(21)-C(20)-H(20)	120.0
C(11)-C(10)-C(15)	119.36(13)	C(19)-C(20)-H(20)	120.0
C(12)-C(11)-C(10)	120.47(12)	C(20)-C(21)-C(16)	120.84(13)
C(12)-C(11)-H(11)	119.8	C(20)-C(21)-H(21)	119.6
C(10)-C(11)-H(11)	119.8	C(16)-C(21)-H(21)	119.6
C(11)-C(12)-C(13)	121.33(13)		

Crystal data and structure refinement for Compound **6**.

Empirical formula	$C_{15}H_9NO$	
Formula weight	219.23	
Temperature	100(2) K	
Wavelength	0.71073 Å	
Crystal system	Monoclinic	
Space group	C2	
Unit cell dimensions	a = 30.5846(19) Å	α= 90°.
	b = 4.7880(3) Å	β= 108.541(2)°.
	c = 14.5788(8) Å	γ = 90°.
Volume	2024.1(2) $Å^3$	
Z	8	
Density (calculated)	1.439 Mg/m^3	
Absorption coefficient	0.091 mm^{-1}	
F(000)	912	
Crystal size	0.410 x 0.110 x 0.020 mm^3	
Theta range for data collection	2.337 to 26.047°.	
Index ranges	-37<=h<=37, -5<=k<=5, -18<=l<=17	
Reflections collected	23927	
Independent reflections	3959 [R(int) = 0.1007]	
Completeness to theta = 25.242°	99.8 %	
Refinement method	Full-matrix least-squares on F^2	
Data / restraints / parameters	3959 / 1 / 307	
Goodness-of-fit on F^2	1.027	
Final R indices [I>2sigma(I)]	R1 = 0.0467, wR2 = 0.1084	
R indices (all data)	R1 = 0.0541, wR2 = 0.1148	
Absolute structure parameter	0.8(10)	
Extinction coefficient	n/a	
Largest diff. peak and hole	0.229 and -0.297 e.$Å^{-3}$	

Crystal data and structure refinement for Compound **7**.

Empirical formula	$C_{21}H_{17}NO$	
Formula weight	299.36	
Temperature	100(2) K	
Wavelength	0.71073 Å	
Crystal system	Triclinic	
Space group	P-1	
Unit cell dimensions	a = 8.5180(8) Å	α= 62.330(4)°.
	b = 10.0713(11) Å	β= 80.923(4)°.
	c = 10.1653(11) Å	γ = 85.320(4)°.
Volume	762.62(14) $Å^3$	
Z	2	
Density (calculated)	1.304 Mg/m^3	
Absorption coefficient	0.080 mm^{-1}	
F(000)	316	
Crystal size	0.100 x 0.050 x 0.029 mm^3	
Theta range for data collection	2.283 to 26.013°.	
Index ranges	-10<=h<=10, -12<=k<=12, -12<=l<=12	
Reflections collected	43967	
Independent reflections	3004 [R(int) = 0.0825]	
Completeness to theta = 25.242°	99.9 %	
Refinement method	Full-matrix least-squares on F^2	
Data / restraints / parameters	3004 / 0 / 208	
Goodness-of-fit on F^2	1.145	
Final R indices [I>2sigma(I)]	R1 = 0.0407, wR2 = 0.1343	
R indices (all data)	R1 = 0.0503, wR2 = 0.1454	
Extinction coefficient	n/a	
Largest diff. peak and hole	0.164 and -0.304 e.$Å^{-3}$	

Crystal data and structure refinement for Compound **8**.

Empirical formula	$C_{21}H_{13}NO$	
Formula weight	295.32	
Temperature	100(2) K	
Wavelength	0.71073 Å	
Crystal system	Monoclinic	
Space group	C2/c	
Unit cell dimensions	a = 23.606(2) Å	α= 90°.
	b = 9.9979(9) Å	β= 118.225(3)°.
	c = 13.7241(13) Å	γ = 90°.
Volume	2853.9(5) $Å^3$	
Z	8	
Density (calculated)	1.375 Mg/m^3	
Absorption coefficient	0.085 mm^{-1}	
F(000)	1232	
Crystal size	0.742 x 0.292 x 0.234 mm^3	
Theta range for data collection	1.958 to 26.019°.	
Index ranges	-28<=h<=29, -12<=k<=12, -16<=l<=16	
Reflections collected	54964	
Independent reflections	2810 [R(int) = 0.0988]	
Completeness to theta = 25.242°	99.9 %	
Refinement method	Full-matrix least-squares on F^2	
Data / restraints / parameters	2810 / 0 / 209	
Goodness-of-fit on F^2	1.070	
Final R indices [I>2sigma(I)]	R1 = 0.0441, wR2 = 0.1175	
R indices (all data)	R1 = 0.0496, wR2 = 0.1257	
Extinction coefficient	0.0045(8)	
Largest diff. peak and hole	0.285 and -0.233 e.$Å^{-3}$	

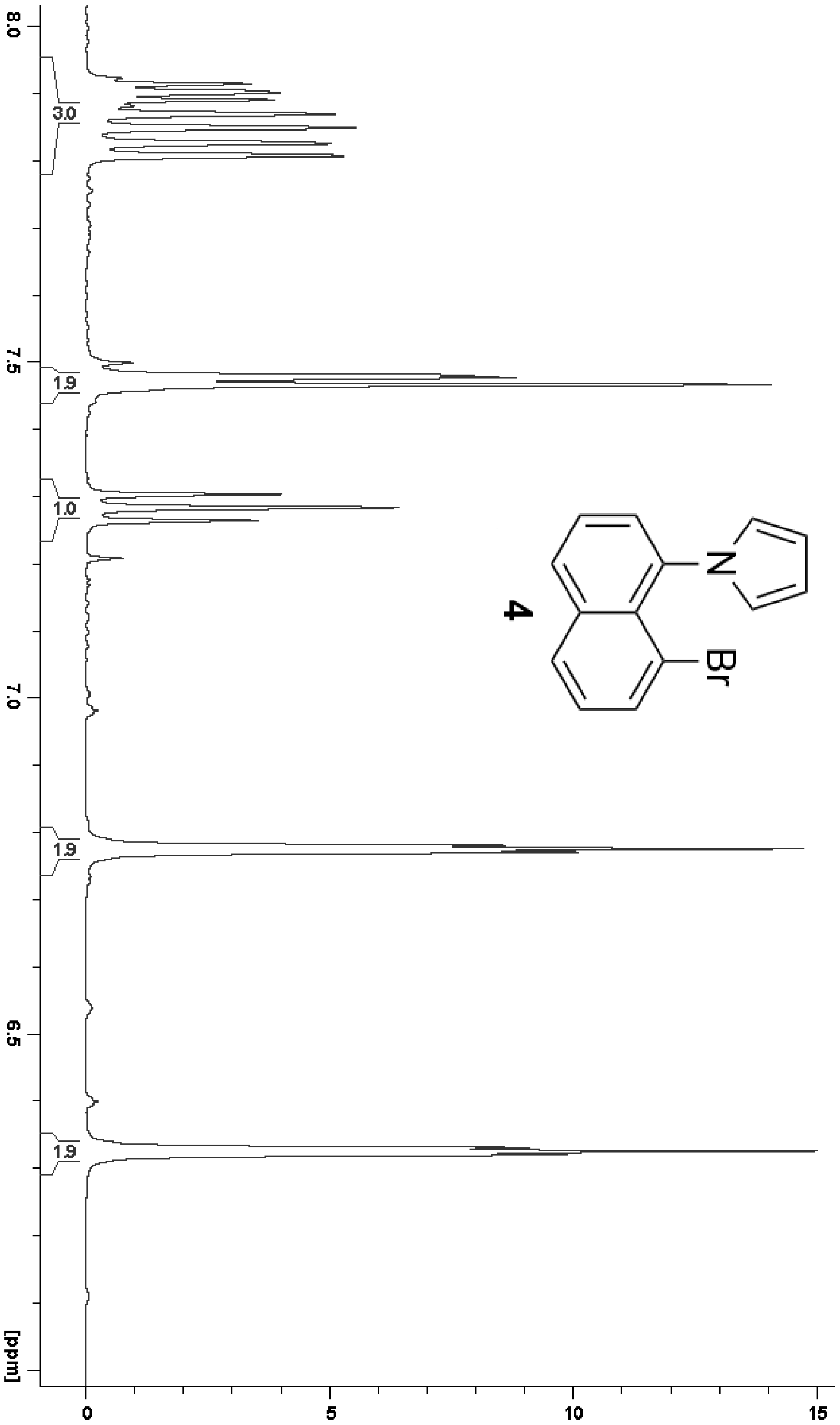

Figure S1. ^{1}H NMR spectrum of **4** in $CDCl_3$.

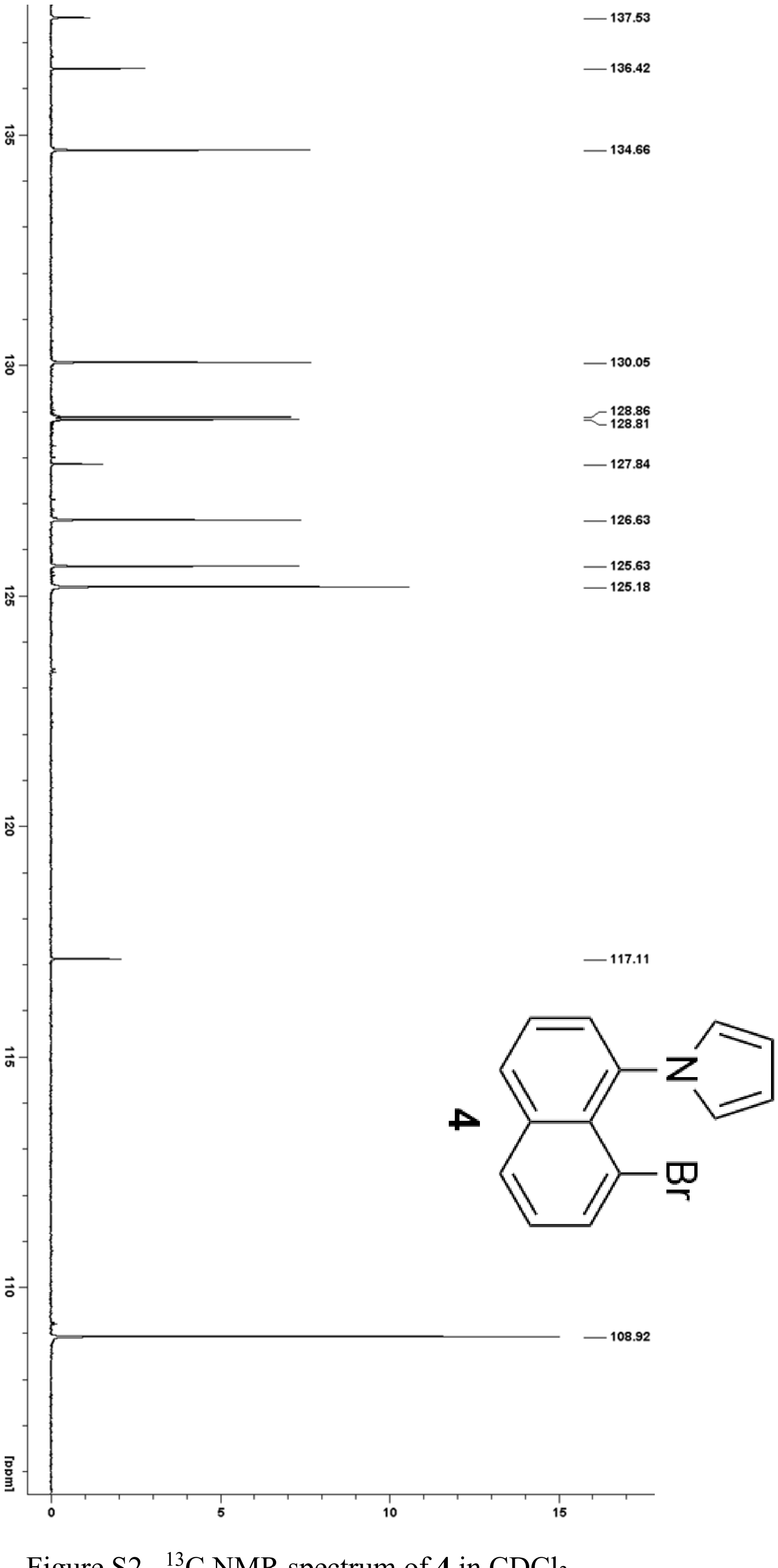

Figure S2. ^{13}C NMR spectrum of **4** in $CDCl_3$.

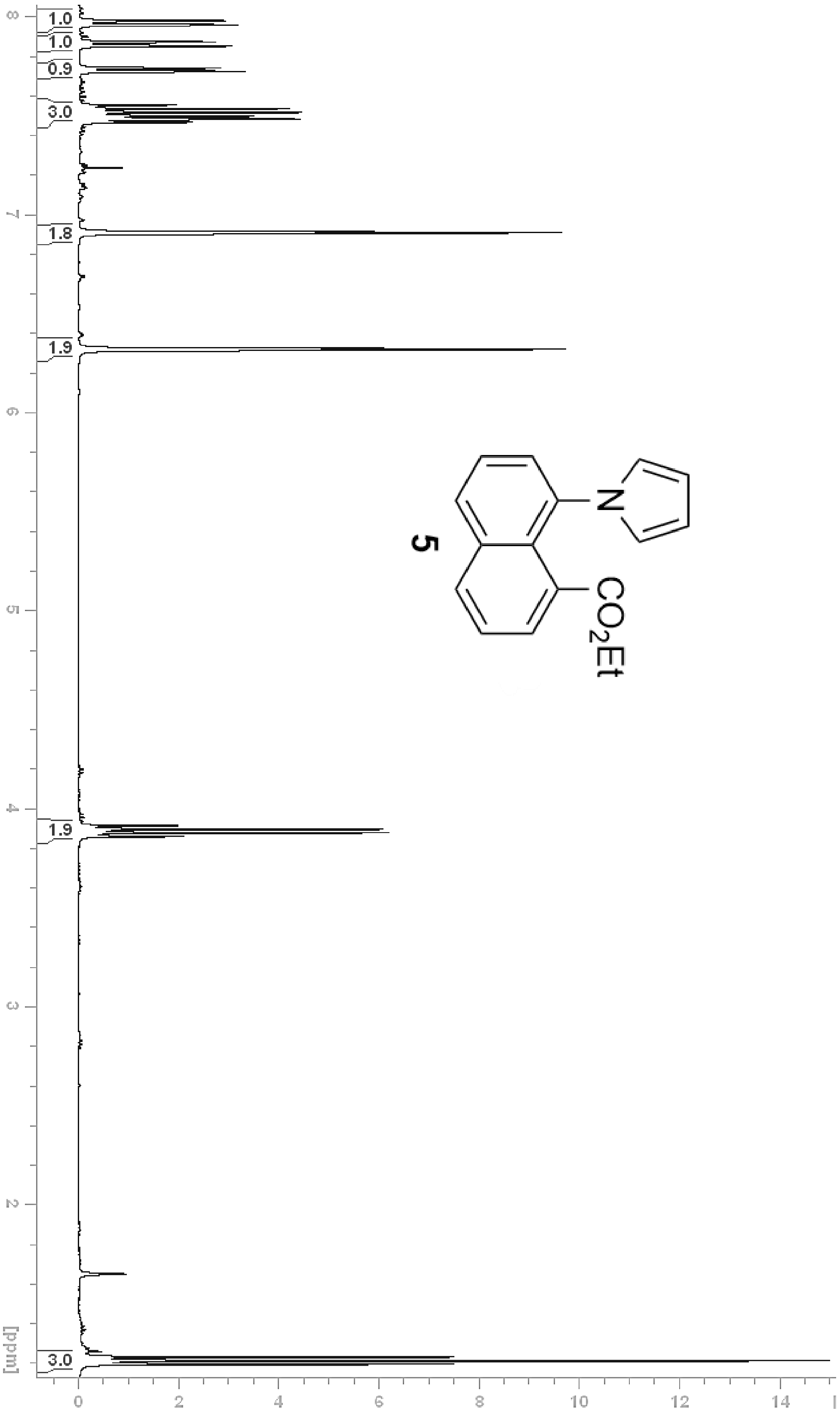

Figure S3. ^{1}H NMR spectrum of **5** in $CDCl_3$.

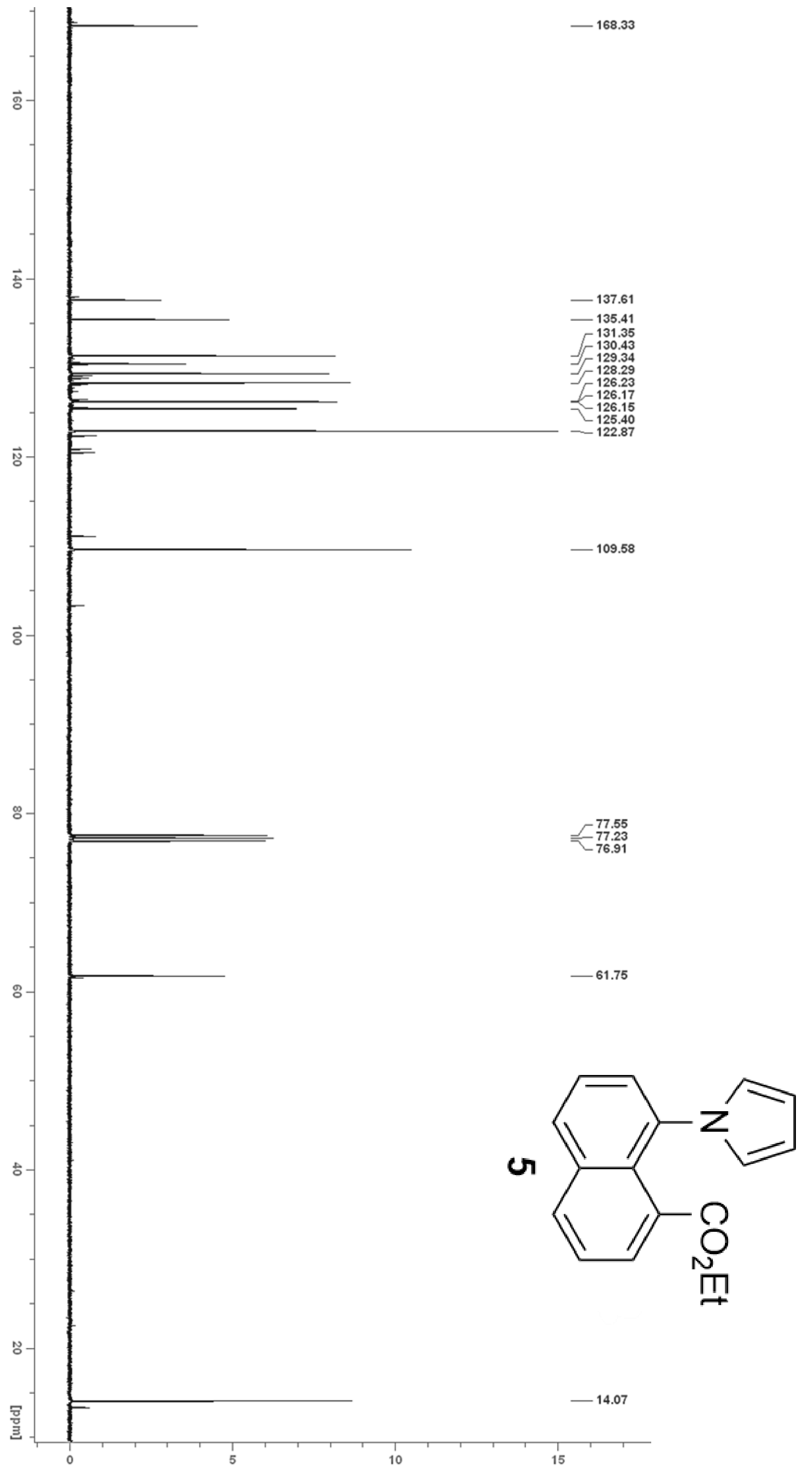

Figure S4. ^{13}C NMR spectrum of **5** in $CDCl_3$.

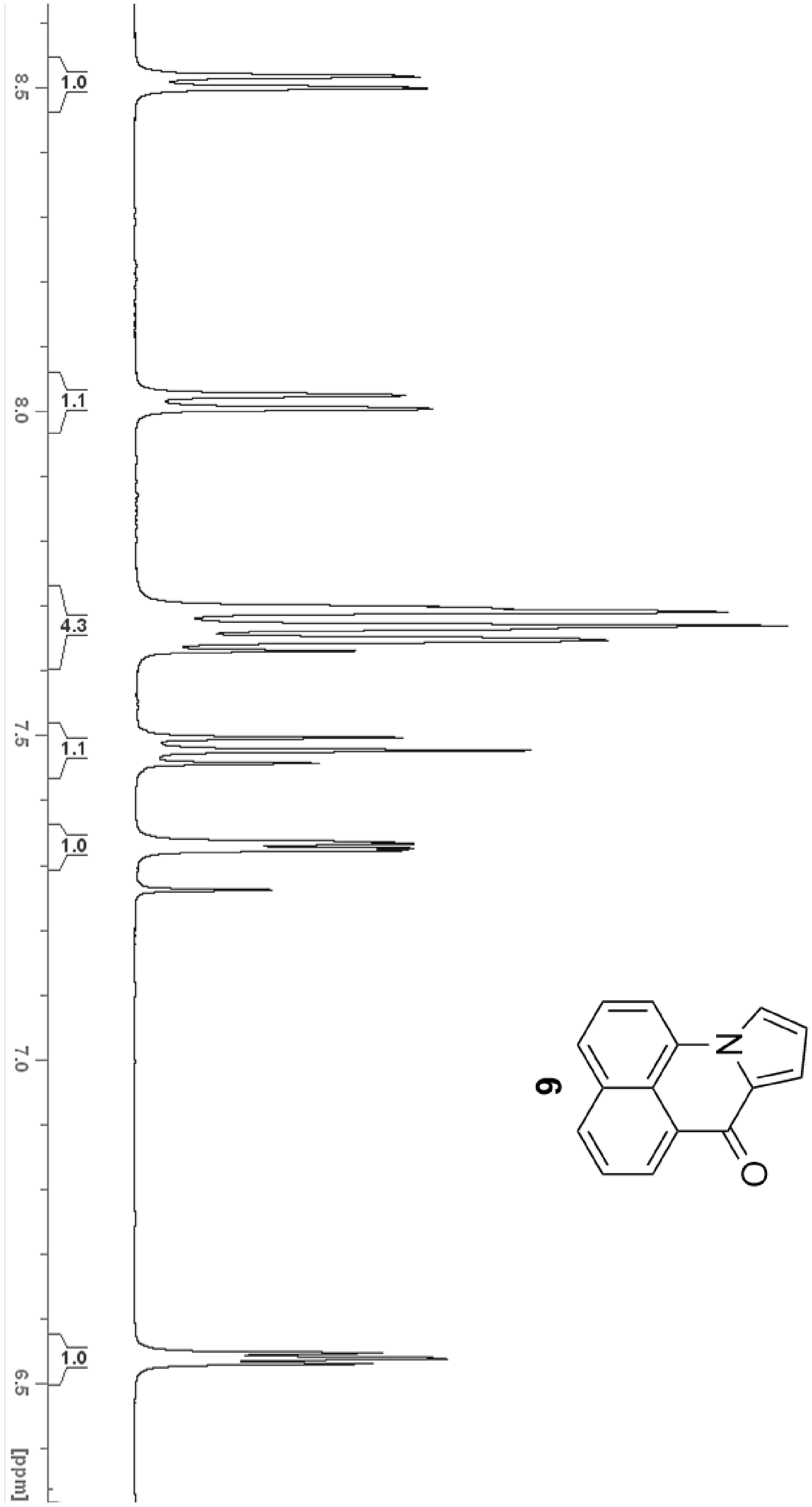

Figure S5. ^{1}H NMR spectrum of **6** in $CDCl_3$.

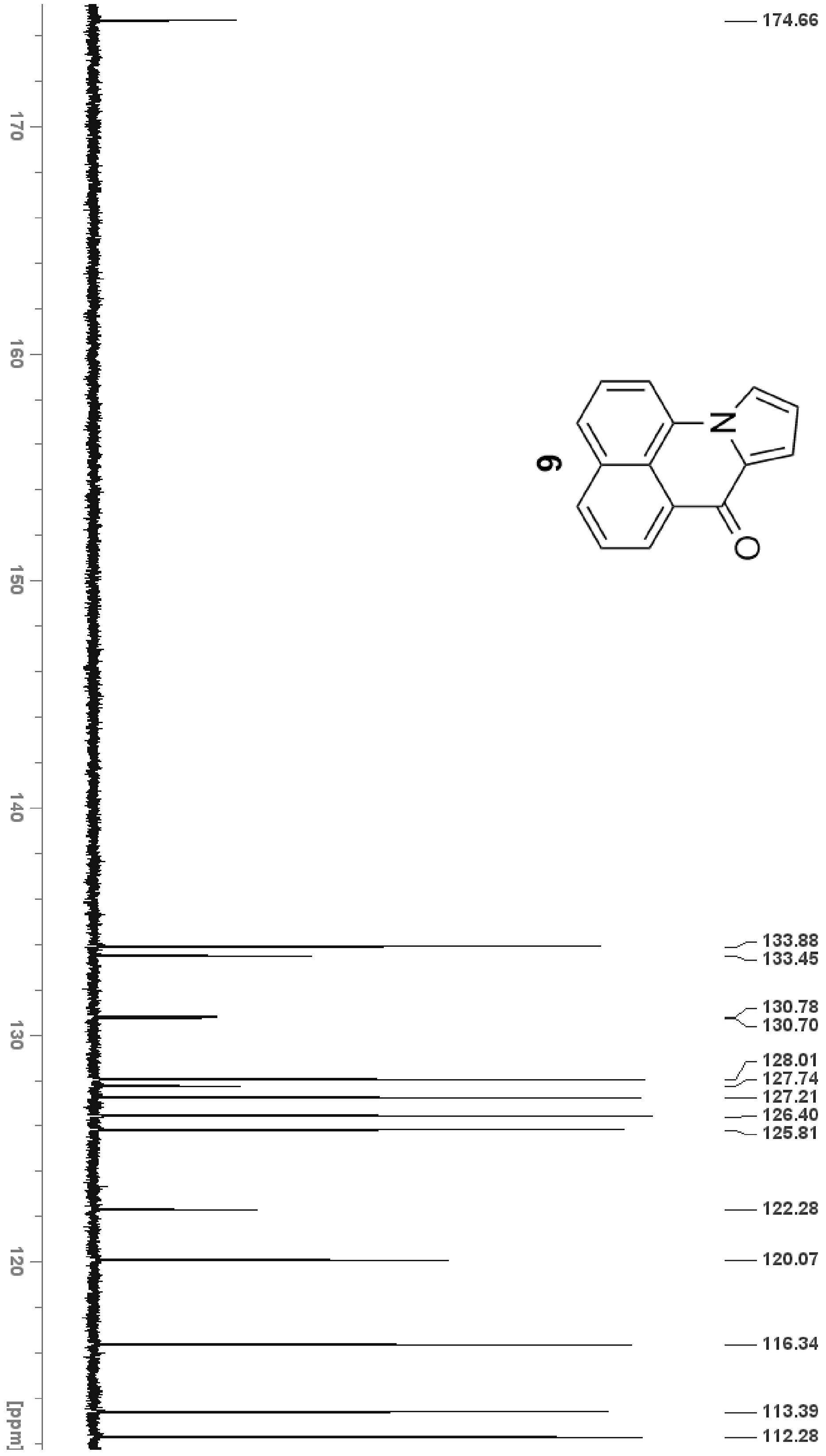

Figure S6. ^{13}C NMR spectrum of **6** in $CDCl_3$.

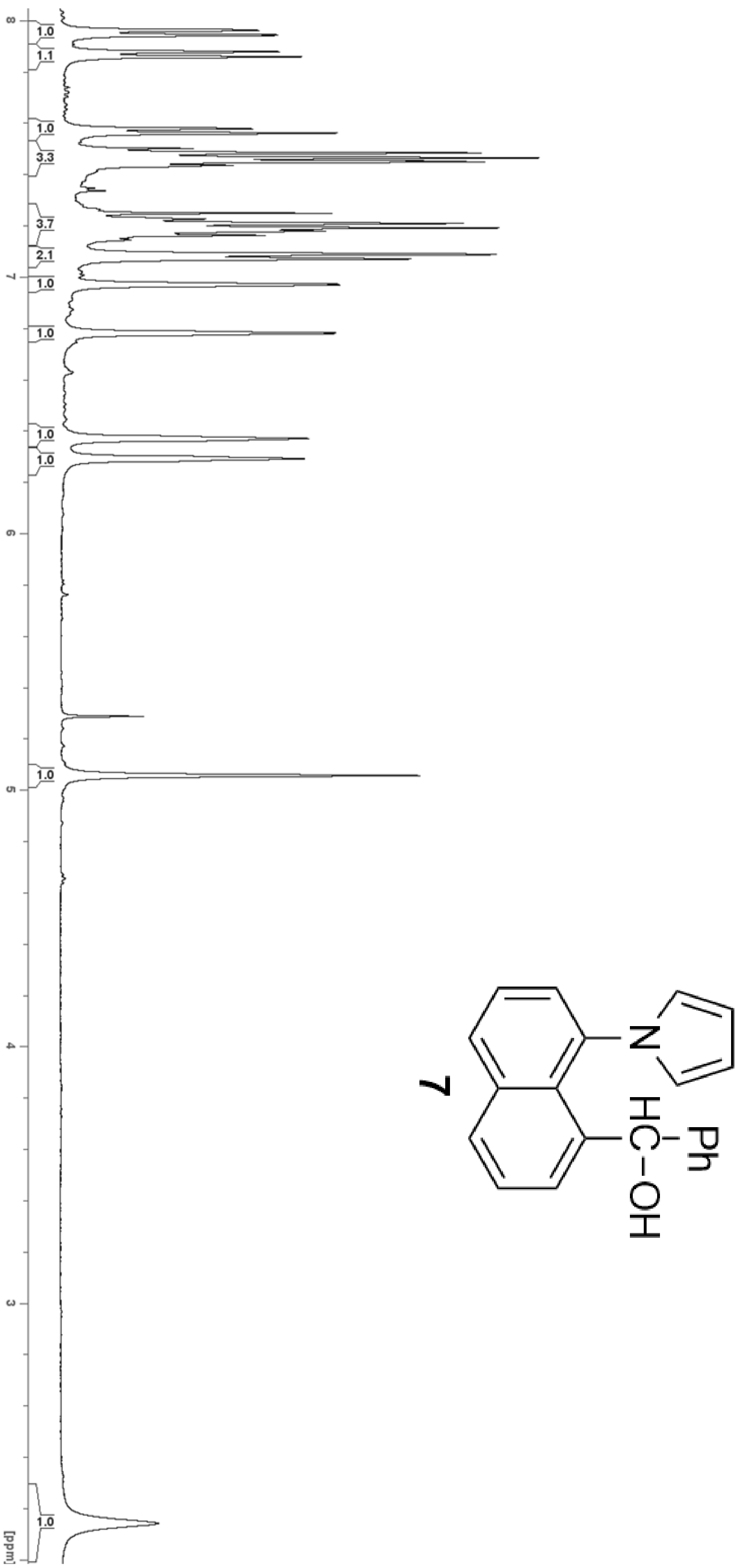

Figure S7. ^{1}H NMR spectrum of **7** in $CDCl_3$.

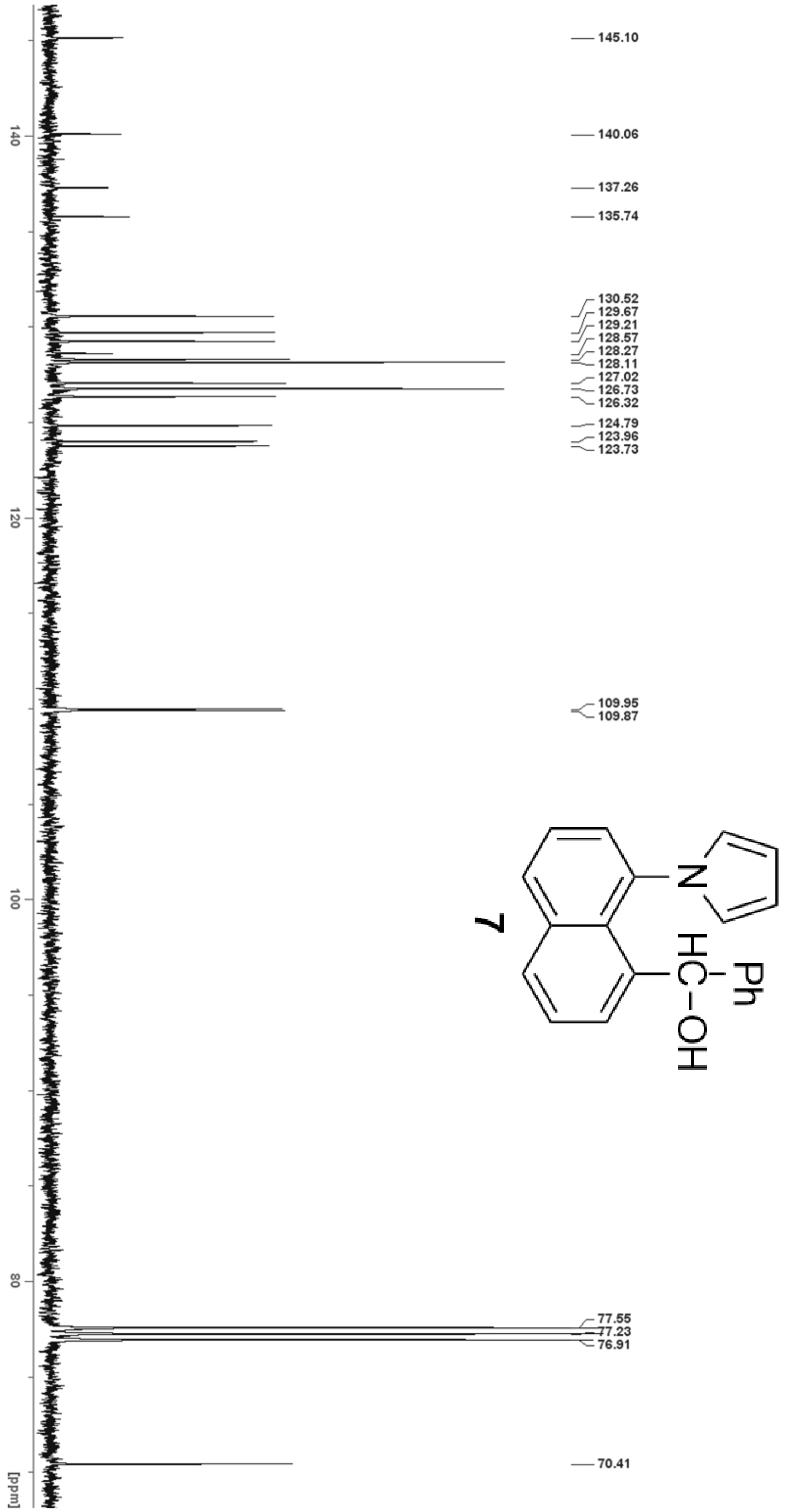

Figure S8. ^{13}CNMR spectrum of **7** in $CDCl_3$.

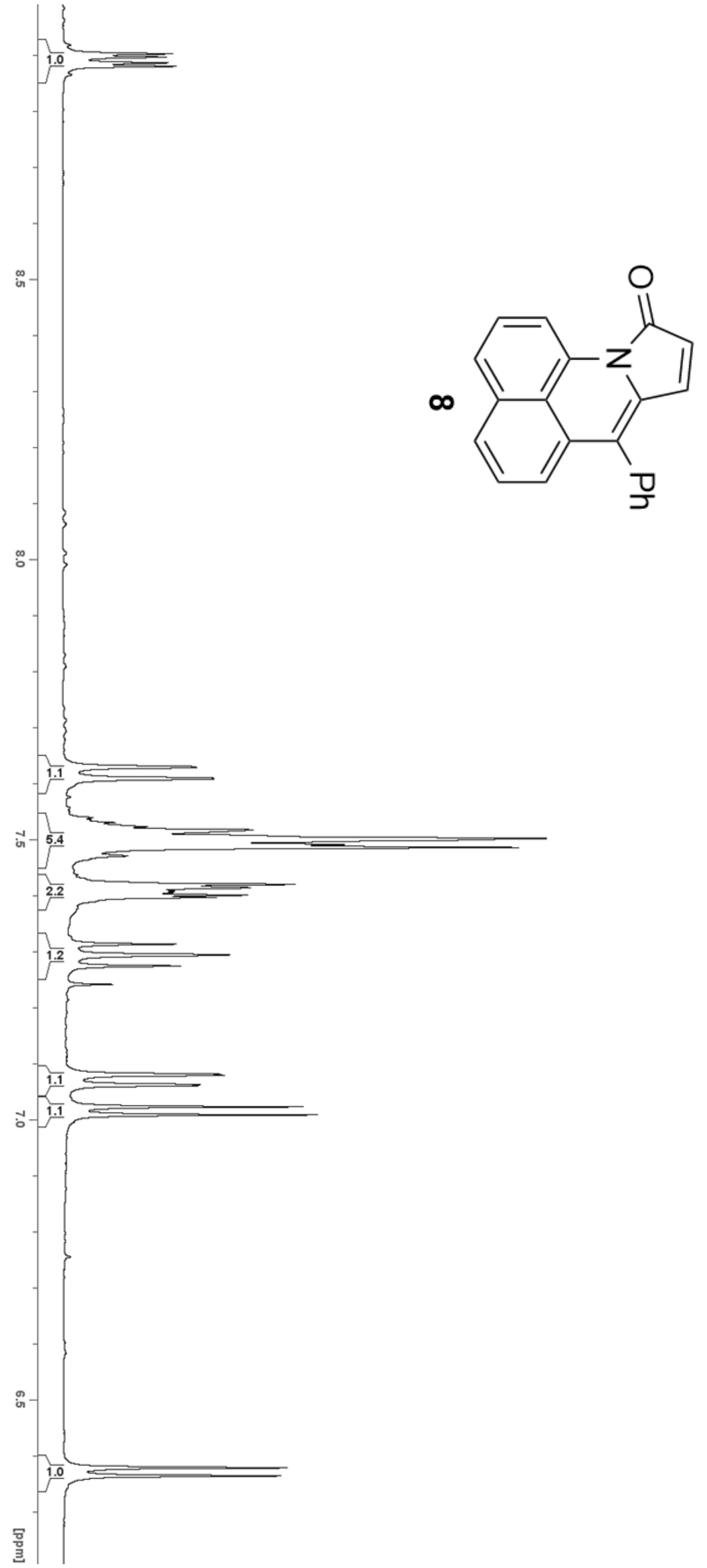

Figure S9. ^{1}H NMR spectrum of **8** in $CDCl_3$.

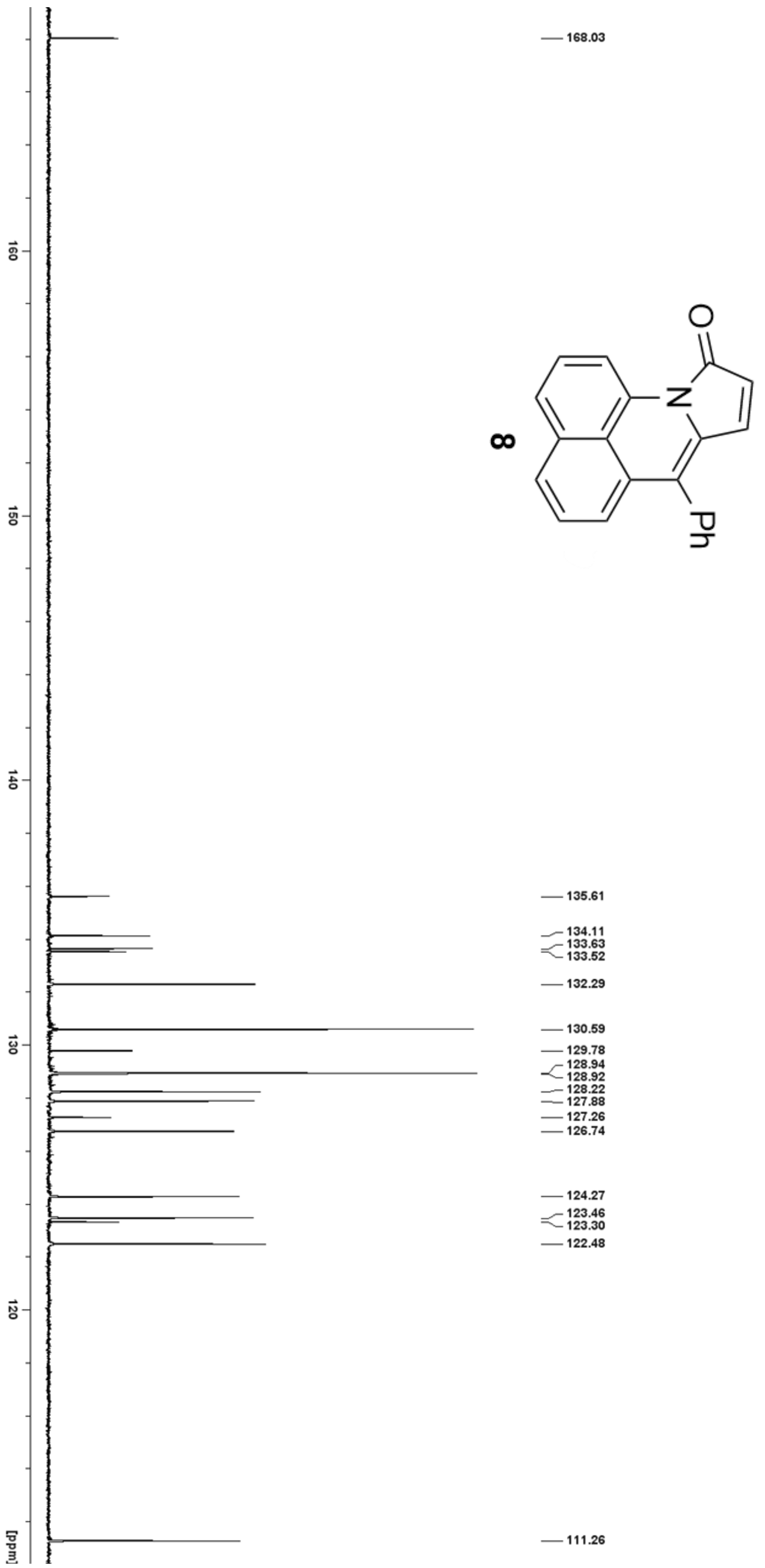

Figure S10. ^{13}C NMR spectrum of **8** in $CDCl_3$.

Table S1. Absorption maxima (nm) and molar absorptivities for **6** and **8** in Figure 4.

solvent	**6**		**8**	
	λ_{max} (nm)	ε (M^{-1} cm^{-1})	λ_{max} (nm)	ε (M^{-1} cm^{-1})
toluene	387	14,900	475	18,900
dichloromethane	388	16,700	471	21,100
acetonitrile	387	16,200	465	19,300
ethanol	392	15,300	469	20,600

Table S2. Fluorescence maxima (nm) and relative integrated areas for **5**-**6** and **8** in Figure 5.

solvent	$E_T(30)$	**5**		**6**		**8**	
		λ_{max} (nm)	int.	λ_{max} (nm)	int.	λ_{max} (nm)	int.
cyclohexane	30.9	469	0.26	460	0.02	590	0.92
toluene	33.9	484	0.53	463	0.04	603	0.78
diethyl ether	34.5	487	0.70	472	0.04	595	0.83
chlorobenzene	36.8	485	0.93	467	0.08	604	0.76
ethyl acetate	38.1	489	0.70	463	0.04	600	0.68
dichloromethane	40.7	489	1.00	474	0.14	600	0.79
acetone	42.2	492	0.66	470	0.11	602	0.71
dimethyl sulfoxide	45.1	499	0.66	485	0.25	612	0.66
acetonitrile	45.6	494	0.63	477	0.21	607	0.65
isopropanol	48.4	494	0.94	496	0.61	595	1.00
ethanol	51.9	496	0.72	501	0.78	596	0.98
methanol	55.4	501	0.83	509	1.00	597	0.75
